# Engineering inertial flow patterns for signal amplification in disc-based protein assays

**DOI:** 10.7150/thno.124439

**Published:** 2026-03-09

**Authors:** Hyun-Kyung Woo, Lauren Philp, Dae-Han Jung, Dominique Zarrella, Yein Chung, Yoonjeong Choi, Jueun Jeon, Hyunho Kim, Cesar M. Castro, Bo R. Rueda, Hakho Lee

**Affiliations:** 1 Center for Systems Biology, Massachusetts General Hospital Research Institute, Boston, MA 02114, USA.; 2 Department of Radiology, Massachusetts General Hospital, Harvard Medical School, Boston, MA 02114, USA.; 3 Vincent Center for Reproductive Biology, Department of Obstetrics and Gynecology, Massachusetts General Hospital, Boston, MA 02114, USA.; 4 Obstetrics, Gynecology and Reproductive Biology, Harvard Medical School, Boston, MA 02114, USA.; 5 Department of Immunology, University of Toronto, Toronto, ON M5S 1A8, Canada.; 6 School of Mechanical Engineering, Korea University, Seoul, 02841, Republic of Korea.; 7 Cancer Center, Massachusetts General Hospital, Harvard Medical School, Boston, MA 02114, USA.

**Keywords:** inertial force, lab-on-a-disc, protein assay, signal amplification, ovarian cancer

## Abstract

Detecting low-abundance proteins is essential in liquid biopsy applications, particularly for extracellular vesicle (EV)-based diagnostics. Here, we introduce RapidEx (*R*otationally *a*mplified *p*rotein *i*ntensity *d*etection of *Ex*tracellular vesicles), a disc-fluidic system engineered for highly sensitive protein assays. RapidEx performs two key functions within a single disc cartridge: i) enzymatic chemical reactions for target labeling and ii) efficient washing to enhance signal-to-background contrast.

**Methods:**

The device leverages a unique interplay between disc-specific inertial forces (centrifugal and Coriolis) and membrane-filter flow dynamics. Unlike conventional systems, RapidEx utilizes a low-permeability membrane to redirect fluid laterally during rotation, thereby enhancing washing efficiency and signal-to-noise ratio. The performance of the device was validated through numerical simulation and tyramide-mediated amplification assays. This system processed plasma samples from patients with ovarian cancer (*n* = 54) and non-cancer controls (*n* = 28), facilitating EV labeling for a panel of cancer markers.

**Results:**

Numerical simulations and experimental validations confirmed that Coriolis-induced lateral flows were found critical to improving labeling and washing efficiency. This synergetic fluidic control with enzymatic chemical reaction enhanced the signal-to-noise ratio, which is >25-fold more sensitive than standard two-step labelling. Applying this platform to clinical samples, protein profiles enabled differentiation between cancer patients and controls and allowed for the identification of platinum therapy resistance in patients prior to chemotherapy treatment.

**Conclusions:**

RapidEx could enable rapid and cost-effective protein-based liquid biopsies, with applications in disease detection and monitoring therapeutic responses.

## Introduction

Proteins are critical cancer biomarkers, whose activity underlies the biomolecular signaling pathways driving cancer pathology 1, 2. Consequently, protein detection can provide key insights into disease classification, treatment response, progression, and emerging resistance mechanisms 3. Extracellular vesicles (EVs), secreted by cells, are known to carry proteins reflective of their cells of origin 4-7. However, detecting EV proteins can be challenging due to the limited protein quantity within EVs as well as the low number of tumor-derived EVs in circulation 8. Recent advances have introduced approaches that enhance analytical sensitivity by engineering signaling moieties, including enzymatic amplification of fluorescent dyes 9, 10 and nanocarrier-mediated labeling 11, 12.

Despite these advances, the clinical translation of EV protein assays remains hindered by several unresolved challenges 13. Many current platforms rely on multistep manual workflows that introduce operator variability and reduce reproducibility 14, 15. Inefficient washing often leads to high background signals, limiting the use of amplification chemistries and compromising analytical specificity 10, 16. These challenges are magnified in busy clinical settings, where assays must be automated and easy to operate to ensure robust performance across technicians and testing sites 17. Furthermore, clinical samples are frequently available only in small aliquots, and tumor-derived EVs are only a minor fraction of total vesicles 18. These constraints demand methods that combine high sensitivity with streamlined, low-volume workflows.

In the face of these limitations, disc-based fluidics could be a promising platform. Disc systems offer precise, programmable fluidic control without external pumps or complex tubing 19, and can be fabricated using thermoplastics for cost-effective, high-throughput manufacturing 20, 21. These attributes are shown to facilitate reproducible, automated, and hands-free operation. Indeed, advanced lab-on-a-disc systems have been implemented for various applications, including immunoassays 22, nucleic acid amplification 23, and tumor-cell detection 24. Nonetheless, relatively few disc systems have been adapted for protein-amplification workflows. A key limiting factor is the requirement for a stringent sample washing to prevent spurious signal amplification, an unmet need for many existing designs.

Herein, we report the development of a disc-based system for enzymatic signal amplification, termed RapidEx (**R**otationally **a**mplified **p**rotein **i**ntensity **d**etection of **Ex**tracellular vesicles). RapidEx performed two key functions within a single disc cartridge: enzymatic chemical reactions for target labeling and efficient sample mixing to enhance signal-to-background contrast. A critical design feature was the integration of disc-specific inertial forces (centrifugal and Coriolis) with a membrane filter system. The filter retained microbeads during the labeling process while the Coriolis force generated sweeping lateral flows for sample mixing and washing. Fluidic simulations elucidated the intricate interplay between membrane flow and Coriolis effect-induced flow, enabling us to optimize design parameters (*e.g.*, membrane permeability, disc rotational speed) to maximize labeling efficacy.

We validated this concept by using our RapidEx prototype. EVs were captured on microbeads, targeted with antibodies, and further subjected to tyramide-assisted signal enhancement. All operations were performed within a single cartridge, offering a consistent and rapid (<1 hour) sample preparation. In a pilot testing, we used RapidEx to process plasma samples of patients with ovarian cancer (*n* = 54) and non-cancer controls (*n* = 28). The RapidEx prototype facilitated the labeling of EVs for a panel of cancer markers (EpCAM, CLDN4, CLDN3, IL6R, STn, CD44, FOLR1, CD24, CA125) and allowed for effective and precise signal amplification. Profiling these markers enabled differentiation between cancer patients and controls, as well as the identification of resistance to platinum therapy in patients prior to chemotherapy treatment.

## Materials and Methods

### RapidEx disc fabrication

The top and bottom layers of the disc were designed using computer-aided design software and fabricated on polycarbonate (PC) sheets (8574K24, 8574K809; McMaster Carr) *via* computer numerical control (CNC) milling (MDX-50 SRP, Roland). Diaphragms were formed at designated valve locations on the top layer by applying encapsulant material (4019862; Ellsworth adhesives), which was cured for 3 hours (20 °C). We separately patterned a pressure-sensitive adhesive (SELECT DF132311; FLEXcon) using a plotter (CE7000-60; Graphtec). We bonded the top and bottom layers using the patterned film as a double-sided adhesive. The interior of the disc was then treated with a 1% Pluronic F-127 solution (0.22 µm filtered, Thermo Fisher Scientific) for 1 hour, followed by washing with distilled water and air-drying. The valve adaptor units (93800A350; McMaster Carr) were aligned with the diaphragms and bonded using epoxy glue (CA-11; 3M), followed by the assembly of valve actuators (91067A114; McMaster Carr) as previously described 25, 26. A track-etched PC membrane (0.2-µm pore size, 13 mm diameter; Millipore Sigma) was inserted into the designated bead chamber through the backside of the disc 24, 25. The quadrant cartridge disc was then assembled on a disc holder. The chambers on the disc were then filled with reagents for the subsequent assays, and the assay chamber was filled with streptavidin (StAv)-coated polystyrene (PS) beads (0.05 mg bead/mL; 50 µL).

### RapidEx disc assay

*Tyramide-amplified bead assay.* **Table S1** shows the assay process in detail. Biotinylated EVs (50 µL; prepared by mixing 10 µL of biotinylated EVs with 40 µL of 0.22-µm-filtered PBS, fPBS) were introduced into the assay chamber and captured on streptavidin-coated 5-µm polystyrene beads (20 min). To promote bead-EV interaction, the disc was operated in one cycle of oscillatory mixing (±120°/s for 30 s), followed by rotational mixing at 600 rpm for 5 min. The beads were then washed with 120 µL fPBS using a spinning step (1800 rpm, 1 min). Primary antibodies (30 µL, 5 µg/mL in 0.1% BSA; **Table S2**) were introduced, and the disc was placed in mixing mode (2 cycles, 10 min). Polymerized horseradish peroxidase (Poly-HRP)-conjugated secondary antibodies (30 µL, 1:2500 in 0.1% BSA) were added next and incubated under the same mixing conditions (2 cycles, 10 min). Both primary and secondary antibody concentrations were selected to ensure about a 10-fold molar excess over the estimated maximum antigen load, thereby supporting reproducible labeling. Following secondary labeling, the sample was washed again with 120 µL fPBS and incubated with the tyramide working solution (1 µL of 100× tyramide and 1 µL of 100× hydrogen peroxide (H_2_O_2_) in 100 µL reaction buffer). The disc was operated in mixing mode for an additional 2 cycles (10 min). A final wash (100 µL fPBS) was performed before transferring the bead-EV complexes to the bead-collection chamber. All incubations were performed at room temperature, as HRP-mediated amplification and antibody binding were empirically found to be robust to modest variations in environmental temperature.

*Two-step antibody bead assay.* The general protocol is the same as described above. Fluorescent-conjugated secondary antibodies (30 µL, 5 µg/mL in 0.1% BSA) were used for the two-step antibody bead assay rather than HRP-conjugated antibodies.

### Bead analysis

The assay-completed disc cartridges were transferred to the jig for a 96-well plate holder. The designated well places were B2 and G11 (compatible with a 96-well plate reading system). Fluorescent signals were read from individual beads (488 nm excitations; 525/40 nm bandpass filters). A total of 5000 events were recorded for each sample. We used FlowJo (v10; BD) for data export. For each target marker (M), we obtained ∆*I*_M_ (*I*_M_ - *I*_IgG_) and used a metric *ξ*_M_ [= ∆*I*_M_ / ∆*I*_CD63_] to normalize marker signals to EV abundance across samples. For microscope image analysis, we imaged labeled beads using a modified Nikon Eclipse Ti2E microscope. Image analyses were performed using ImageJ.

### Model for microfluidic systems and numerical simulation implementation

In this system, in addition to the gravitational force, centrifugal force, and Coriolis force were considered as additional body forces. We neglected the Euler force since the disc was assumed to rotate at a constant rotational speed. We used a Cartesian coordinate system to describe the disc rotating clockwise around the *z*-axis. In terms of the Cartesian velocity components, *u_x_*, *u_y_*, and *u_z_*, defined along each *x*, *y*, and *z* coordinate axis, the gravitational (*f*_g_), centrifugal (*f*_cen_), and Coriolis force densities (*f*_cor_) can be expressed as:



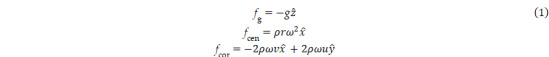



where *g*, *ω*, *ρ* are the gravitational constant, angular velocity, and liquid density, respectively, and *r* is the distance of a liquid element from the rotation center. Although the gravitational force was included in the model, its magnitude was considerably smaller than that of the centrifugal and Coriolis forces; therefore, it had only a limited effect on the overall flow behavior.

Due to the low Reynolds number in the microfluidic device, the laminar flow was assumed. The flow is governed by the Navier-Stokes equation for a steady, incompressible Newtonian fluid. A no-slip boundary condition was imposed on the confining sidewalls. For the inlet and outlet boundary conditions, we applied the volumetric flow rate as given by the following equation:



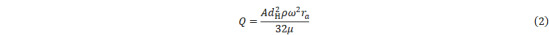



where *A* is the channel's cross-sectional area, *d*_H_ is the hydraulic diameter, *r*_a_ is the mean distance of the fluid elements from the center of the rotating disc, and *μ* is the dynamic viscosity of the fluid. Eq. (2) was derived from the Hagen-Poiseuille equation considering centrifugally induced pressure when the laminar flow is fully developed in the microchannel 27, 28. In the experiment, because the capillary valve does not operate at lower speeds (below 600 rpm), the volumetric flow rate could not be experimentally determined at these lower rotations. Therefore, we used Eq. (2) to encompass a wider range of rotational speeds. To ensure that the total mass of the system remains constant over time, the volumetric flow rate injected at the inlet and the flow rate exiting through the outlet are intended to be the same. For this purpose, an open boundary condition is necessary, which is intended to be applied to a boundary with a narrow cross-sectional area to suppress backflow. Based on this intention, the volumetric flow rate at the outlet was hypothetically characterized using Eq. (2), while an open boundary condition was applied at the inlet, given that the narrow structure of the inlet channel locally suppresses backflow. For numerical simulation studies based on this model, we utilized the Free and Porous Media Flow module provided in COMSOL Multiphysics software (COMSOL Inc., USA).

### Cell lines and culture conditions for EV isolation

The ovarian cancer cell lines used in this work (OV90, CaOV3, and SKOV3) were obtained from the American Type Culture Collection. CaOV3 and SKOV3 were grown in Dulbecco's modified Eagle medium (Thermo Fisher Scientific), whereas OV90 was cultured in RPMI-1640 medium (Thermo Fisher Scientific). Each medium was supplemented with 10% fetal bovine plasma (FBS, Thermo Fisher), along with 100 U/mL penicillin and 100 µg/mL streptomycin (Gibco). Cells were cultured at 37 °C in a humidified incubator with 5% CO_2_. For EV harvesting, cells were incubated in a medium with 1% exo-free fetal bovine plasma (Thermo Fisher Scientific) for 48 h. Approximately 100 mL of conditioned medium was then collected and centrifuged (300 × g, 10 min), followed by a second spin at 2000 × g for 10 min. The resulting supernatants were passed through 0.22-µm membrane filters (cat# 430767, Corning), and the filtrates were concentrated using centrifugal filter units (10 kDa cutoff; Centricon-70, Merck Millipore) at 3,500 × g for 30 min at 4°C. Size-exclusion chromatography (SEC) was subsequently used to isolate EV from the concentrated medium. First, an SEC column was prepared by packing 10 mL of prewashed Sepharose CL-4B (GE Healthcare) into a 10 mL syringe (BD Biosciences) lined at the bottom with a nylon mesh (11 µm pore size; NY1102500, Millipore Sigma). Columns were allowed to settle for 24 hours and stored at 4 °C. Prior to loading samples, each column was rinsed with 10 mL of fPBS. Then, the concentrated media sample was applied to the column, and the 7th to 10th fractions were collected (1 fraction = 0.5 mL). A total of 2 mL of these pooled fractions was then concentrated using an Amicon Ultra-2 Centrifugal Filter (10 kDa cut off; Millipore Sigma).

### Transmission electron microscopy (TEM)

Negative staining was performed following a standard adsorption and staining procedure. A strip of parafilm was secured to the bench with a drop of water, and all incubations were carried out on the clean parafilm surface. Carbon-coated copper grids (CF400-CU, Electron Microscopy Sciences) were rendered hydrophilic by glow discharge (25 mA, 20 s) immediately prior to use. A sample (5 µL) was adsorbed to the grid for 1 min, after which excess liquid was blotted using filter paper (Whatman #1). The grid was briefly floated on a water drop for washing, blotted again, and stained with 1% uranyl acetate (#22400, Electron Microscopy Sciences) for 30 sec. Excess stain was gently removed using filter paper. The prepared sample was imaged using a JEOL 1200EX TEM equipped with an AMT 2k CCD camera. For immunogold labeling, an EV sample (5 µL) was adsorbed on glow-discharged grids (5 min) and blocked with 1% BSA (10 min). Grids were incubated with mouse anti-human CD63 antibody (1:30, #556019, BD Biosciences) for 30 min, washed in PBS, and incubated with rabbit anti-mouse secondary antibody (1:50, #ab6709, Abcam) for 30 min. After PBS washing, the grids were incubated with 10-nm gold nanoparticles conjugated with protein A (University Medical Center Utrecht) for 20 min. After final PBS and water washes, samples were stained with 1% uranyl acetate and imaged.

### Nanoparticle tracking analysis (NTA)

We used a NanoSight LM10 system (Malvern). Samples were diluted in fPBS to yield 25-100 particles per frame. For each sample, three 30-second videos were acquired and processed using NTA software (version 3.2) with a detection threshold set to 3.

### Enzyme-linked immunosorbent assay (ELISA)

For the CD63 ELISA, capture antibodies against CD63 (4 µg/mL; #215-020, Ancell) were added to a 96-well plate (50 µg/well, Nunc MaxiSorp flat-bottom, Thermofisher) and incubated overnight at 4 °C. The wells were rinsed with a 200-µL wash buffer (PBS with 0.1% BSA) and then blocked with 1% BSA (200 µL/well) for 2 hours (20 °C). After another wash step, diluted EV samples were added (50 µL/well) and incubated for 2 hours at 20 °C. Following two additional washes, biotinylated anti-CD63 antibodies (500 ng/mL, #215-030, Ancell; 50 µL/well) were added and incubated for 1 hour (20 °C). After washing twice, HRP-conjugated streptavidin (1:20,000 diluted in 0.1% BSA; #405210, BioLegend) was added (50 µL/well) and incubated for 20 min (20 °C). The wells were then triple-washed before adding 100 µL of 3,3',5,5'-tetramethylbenzidine (BioLegend). The reaction proceeded for 30 min (20 °C) and was quenched by adding the stop solution (50 µL). Absorbance at 450 nm was measured using a Tecan plate reader.

### Western blot

EVs were enriched from the plasma and lysed in 10× RIPA buffer (Abcam) for 30 min on ice. The lysate was then centrifuged at 14,000 × *g* (10 min, 4 °C), and the clarified supernatant was collected. Protein levels were quantified using a BCA assay (Thermo Fisher Scientific), after which the samples were resolved on a 10% SDS-polyacrylamide gel. The primary antibodies used were Alix (1:1,000, JM85-31, Invitrogen), biotinylated CD63 (1:1,000, AHN16.1/46-4-5, Ancell), and histone H2B (1:1,000, D2H6, Cell Signaling Technology). We biotinylated the Alix and histone H2B antibodies (sulfo-NHS-biotin; Thermo Fisher Scientific) and generated signals using HRP-conjugated streptavidin (1:2,000, R&D Systems).

### Clinical samples

The study was conducted under approval by the Institutional Review Board (IRB) of Dana-Farber/Harvard Cancer Center (IRB number 07-049), and all procedures adhered to the respective institutional guidelines. Plasma samples were collected from control groups (*n* = 28) and patients with ovarian cancer (*n* = 54) who had provided written consent through the Massachusetts General Brigham Biobank and our institutional gynecologic tissue repository. Patients with FIGO stage IIIC and IV HGSOC who underwent primary cytoreductive surgery and had available preoperative plasma samples were identified from the repository. Clinical data, including age, date of diagnosis, pre-operative CA-125, histology, stage and treatment, and recurrence data, were obtained from electronic medical records. Patients were classified as platinum-sensitive or platinum-resistant based on standard definitions 29. After cohort selection, pre-treatment plasma samples from each patient were obtained from the sample repository and assigned study ID numbers, which were used to blind investigators to clinical data during subsequent analyses.

### Patient sample processing

EVs were isolated from patient plasma samples using SEC columns (qEVoriginal, 70 nm; Izon). A 500 µL plasma input yielded ~250 µL of concentrated EVs following centrifugation with an Amicon Ultra-2 filter (10 kDa cutoff; Merck Millipore). EV samples were subjected to multifactored characterization. Total protein analysis (**Figure S1A**) indicated that the samples contained a sufficient amount of protein, whereas nanoparticle tracking analysis (**Figure S1B**) showed that the majority of isolated particles fell within the expected EV size range (50-300 nm). Furthermore, Western blot analysis (**Figure S1C**) revealed strong expression of a canonical EV marker (CD63) with negligible levels of the non-EV marker histone H2B, indicating minimal contamination by cellular debris or other non-EV particles. For biotinylation, isolated EVs were incubated with 1 µL of 10 mM EZ-Link Sulfo-NHS-LC-Biotin (Thermo Fisher Scientific) for 30 min at 20 °C, and excess biotin was removed by twice PBS washes using an Exodisc device 21, yielding ~200 µL of biotinylated EVs. For downstream RapidEx analysis, 10 µL of the eluted sample was used per marker set. Three RapidEx discs were employed to analyze a total of 11 markers: nine ovarian cancer-associated markers, CD63 (to confirm EV presence and quantify EV input), and an isotype IgG control. Each disc accommodated four markers simultaneously, and the same protocol was applied uniformly across all discs.

### Statistics

We used R version 4.4.1 for statistical analysis. Profiling data, comprising expression levels of 10 markers per sample, were imported. Samples were stratified into four comparison groups: healthy donor (HD), early-stage (ES) ovarian cancer (OvCa), late-stage platinum-sensitive (PS) OvCa, and late-stage platinum-resistant (PR) OvCa. Normalization was performed using CD63, an established EV marker, followed by z-score transformation to standardize features to a mean of zero and unit variance. To identify predictive markers for OvCa diagnostics, logistic regression analysis was employed. Risk scores were computed from regression coefficients, and receiver operating characteristic (ROC) curves were constructed. The area under the curve (AUC) served as a measure of predictive accuracy. Linear Discriminant Analysis (LDA) was utilized to assess group separability and visualize decision boundaries among the four distinct cohorts (HD, ES, PS, and PR). This analysis involved data preprocessing, model fitting, generating decision boundaries, visualizing results, and calculating a confusion matrix. The overall performance of the LDA model was evaluated using leave-one-out cross-validation (LOOCV). This iterative process involved sequentially excluding one sample, training the model on the remaining data, and assessing the prediction accuracy on the excluded sample. This procedure was repeated until each sample had served as the validation set. Standard errors for the LOOCV-derived prediction accuracy rates were estimated using a bootstrap method with 1000 iterations.

## Results and Discussion

### RapidEx disc design

**Figure 1:**
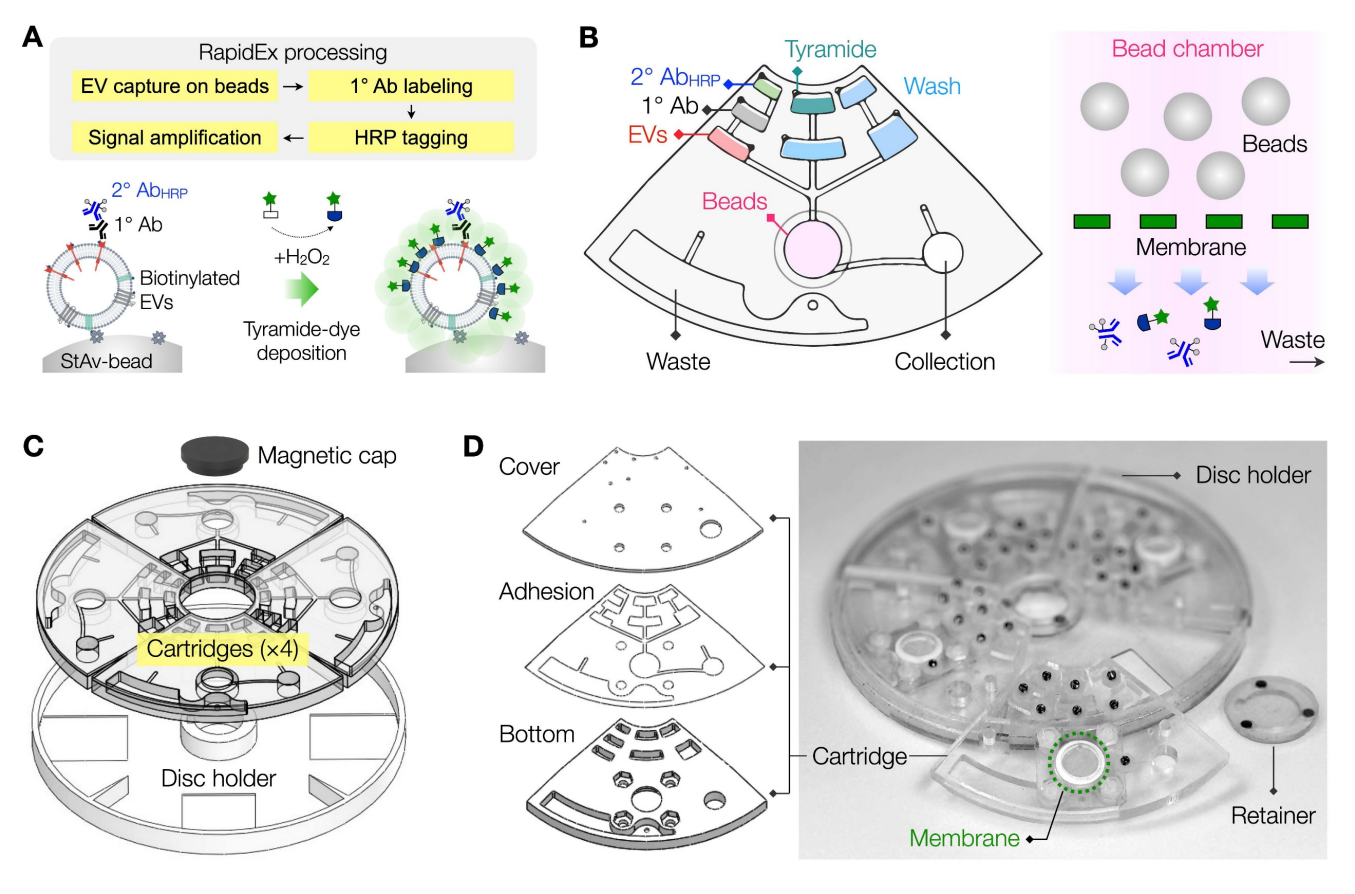
**Overview of the RapidEx approach. (A)** Schematic of the EV assay workflow using RapidEx. Biotinylated EV samples were introduced and captured on streptavidin (StAv) coated microbeads. Target EV proteins were labeled sequentially with a primary antibody (1°Ab), and a polymerized horseradish peroxidase (poly-HRP)-conjugated secondary antibody (2°Ab_HRP_). Upon addition of tyramide-dye and hydrogen peroxide (H_2_O_2_), the HRP catalyzed the dense deposition of activated dye on the bead surface, achieving signal amplification. **(B)** Scheme of the RapidEx device. An integrated centrifugal device was designed to perform RapidEx processing steps: EV capture on beads, EV protein labeling, and signal amplification. In the assay chamber integrated with a membrane, residual 2° Ab_HRP_ and tyramide reagents were effectively removed through washing. **(C)** Quadrant cartridges were assembled on the disc holder with a magnetic cap. **(D)** Scheme of the cartridge layers and photograph of a RapidEx device. The compact disc-sized device (diameter of 11.4 cm) consisted of machined plastic layers bonded with a thin (35 µm) adhesive layer.

**Figure 1A** shows the process performed in RapidEx for amplified EV-protein detection. Biotinylated EVs were captured on StAv-coated microbeads; these beads served as a solid substrate for sample processing. Captured EVs were labeled with primary antibodies (1° Ab) for target proteins, followed by an oxidizing enzyme (poly-HRP) through secondary antibodies (2° Ab_HRP_). Subsequently, the beads were mixed with tyramide-dye conjugates and H_2_O_2_. The HRP-labeled EVs catalyzed the generation of tyramide radicals, resulting in the dense deposition of fluorescent dyes for signal amplification. Finally, the prepared samples were retrieved from a RapidEx device and analyzed *via* flow cytometry (FCM) to quantify the fluorescence of individual beads.

We designed the RapidEx disc to simplify the assay process. A quadrant cartridge was designed, incorporating chambers for samples, microbeads, and reagents (**Figure 1B**). The bead chamber contained a membrane filter at its bottom, enabling the removal of excess reagents during wash steps (see **Figure S2** for detailed processing steps). Following the labeling, the EV-bead samples were collected in a designated chamber for FCM detection. Four individual cartridges were mounted onto a circular holder and secured magnetically, forming a single, integrated disc (**Figure 1C**). The final assembled disc measured 12 cm in diameter.

We fabricated disc cartridges using plastic materials (**Figure 1D**). Fluidic structures were precision-milled on polycarbonate sheets, producing a top cover plate (1.6 mm thick) and a bottom chamber plate (4.5 mm thick). These plates were bonded together using a thin (35 µm) pressure-sensitive adhesive layer. A membrane filter was then attached through the top cover. Torque-activated valves were subsequently installed on the top plate. To operate the RapidEx disc, we utilized our custom-designed spinner (**Figure S3A**), which controlled disc rotation according to user input. After sample processing, quadrant cartridges were detached and placed on an FCM jig. Through this jig, the cartridges could be loaded into the 96-well plate holder in an FCM machine, eliminating the need for additional sample transfer steps (**Figure S3B**).

### Simulation of fluidic profile within RapidEx

**Figure 2:**
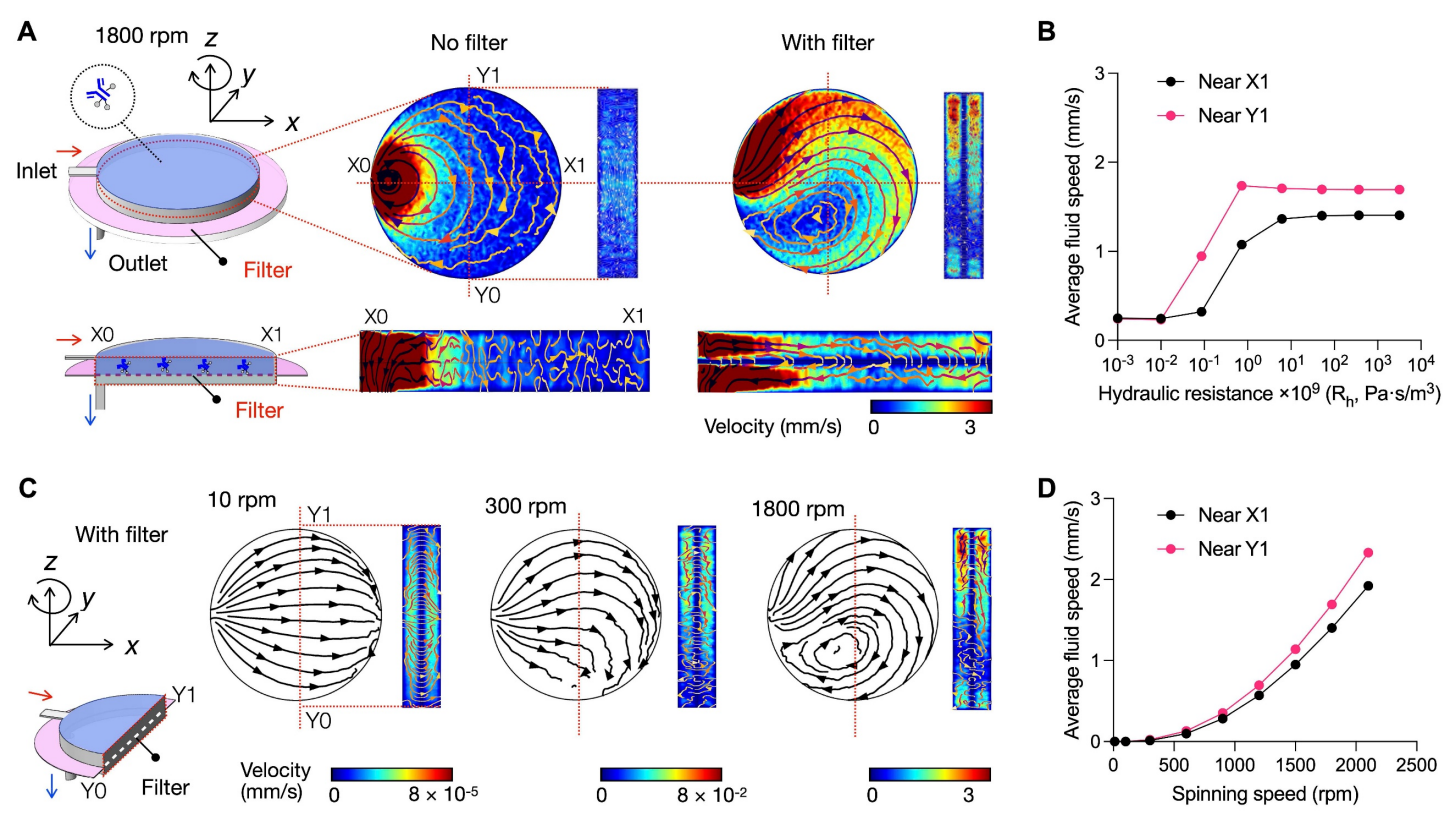
** Simulation of fluidic profile in RapidEx.** A right-handed coordinate system was defined at the disc center: the z-axis aligned with the axis of disc rotation, the x-axis oriented radially through the center of the assay chamber, and the y-axis positioned perpendicularly to both. The disc rotated clockwise around the z-axis. X1 and Y1 represent the far ends of the assay chamber along the x- and y-axes, respectively. **(A)** Influence of a filter in the bead assay chamber spinning at 1800 rpm. Three different cross-section views (*x-y*,* x-z*, and y-z planes) were shown. Without a filter, the flow entering through the inlet channel follows the shortest path to the outlet channel (at X0). With a filter present, the entering flow encountered resistance, redirecting the fluid from the shortest path to travel toward X1. The deviated flow stream became more clearly visible near the Y1 wall region in the *y*-*z* cross-section view. **(B)** Influence of the filter's hydraulic resistance (R_h_) on average velocity. As the resistance increased, the average fluid speed at the reference points (X1 and Y1) near the edge of the assay chamber also increased. **(C)** Effect of spinning speed on fluidic profile with the filter system. At a low speed of 10 rpm, the flow maintained a symmetric profile along the x-axis. As the spinning speed increased, the fluidic flow progressively shifted toward the x-axis, becoming less symmetric. **(D)** The average fluid speed at the reference points (X1 and Y1) increased with higher spinning speed.

We performed numerical simulations to guide disc design and operation, specifically by calculating velocity profiles within the assay chamber. For this analysis, a right-handed coordinate system was defined at the disc center (**Figure 2A**, left): the *z*-axis was aligned with the axis of disc rotation (clockwise), the *x*-axis was oriented radially through the center of the assay chamber, and the *y*-axis was positioned perpendicular to both. Two reference points, X1 and Y1, were designated at the far ends of the assay chamber along the *x*- and *y*-axes, respectively.

First, we analyzed the influence of a filter on the fluidic flow. In the absence of a filter (**Figure 2A**, middle), the input flow was predominantly channeled to the immediate outlet (see the time-lapse movie in **Movie S1**), resulting in a low flow pattern across the assay chamber. In contrast, with a filter present (**Figure 2A**, right), the fluidic flow was redirected, following the lateral pressure gradient induced by centrifugal and Coriolis forces. The radial flow (**Figure 2A**, right bottom) was largely induced by the centrifugal force; the transverse flow in the y direction (**Figure 2A**, right top) was caused by the Coriolis force. Time-lapse movie (**Movie S2**) corroborated these observations, demonstrating persistent lateral flow deviation and sustained asymmetry in the velocity distribution.

We monitored the flow patterns near the edge of the assay chamber to evaluate the influence of the filter's hydraulic resistance (*R_h_*). Specifically, we measured and averaged the fluidic speed within an upper chamber, defined by the filter and a vertical plane located 10% of the chamber diameter from the X1 and Y1 reference points. As the filter**'**s *R_h_* increased, the average fluid speed at these edge points also increased (**Figure 2B**), confirming that the filter significantly influences lateral flow deviation.

Next, we examined the influence of rotational speed on fluidic flow (**Figure 2C**). At a low rotational speed (10 rpm), the flow pattern was symmetric along the *x*-axis, indicating a minimal effect of the Coriolis force. As the speed increased (300 rpm), the fluidic flow deviated from the *x*-axis, following the lateral pressure gradient induced by Coriolis forces. At higher rotational speed (1800 rpm), the flow exhibited asymmetric velocity distribution and vortex formation, which became pronounced under the influence of the Coriolis force. Overall, higher rotational speed enhanced the flow along the chamber sidewalls, as evidenced by the increased average velocities at X1 and Y1 points (**Figure 2D**). Additional simulations confirmed that this enhancement was more pronounced with increasing radial distance of the assay chamber from the rotation center (**Figure S4**), consistent with theoretical predictions of the Coriolis effect.

We further generalized the flow behavior using the Ekman number (*E_k_*), a dimensionless parameter that captures the balance between viscosity and Coriolis-induced inertial effects. Simulations revealed that increasing fluid viscosity at a fixed rotational speed raised *E_k_* and weakened the lateral deviation of the flow, whereas proportionally increasing the rotation speed to maintain a constant *E_k_* restored the original flow pattern (**Figure S5**). These results indicate that Coriolis-driven lateral flow is governed not by viscosity or rotation alone, but by their combined ratio expressed in *E_k_*, providing a simple design criterion for ensuring robust lateral flow in disc-based fluidics (see **Supplementary Note** for details).

### Sample washing driven by the Coriolis effect

We experimentally evaluated the washing efficiency of the RapidEx device. We varied filtering conditions, using track-etched polycarbonate (TEPC) membranes (800 nm or 200 nm pores), anodic aluminum oxide (AAO) membranes (20 nm pores), and a no-filter control (see **Table S3** for a comparison of filter hydraulic resistances). Washing performance was quantified by introducing a colorimetric dye solution into the assay chamber and measuring residual dye intensity following the washing procedure; lower intensity indicated higher efficiency.

**Figure 3:**
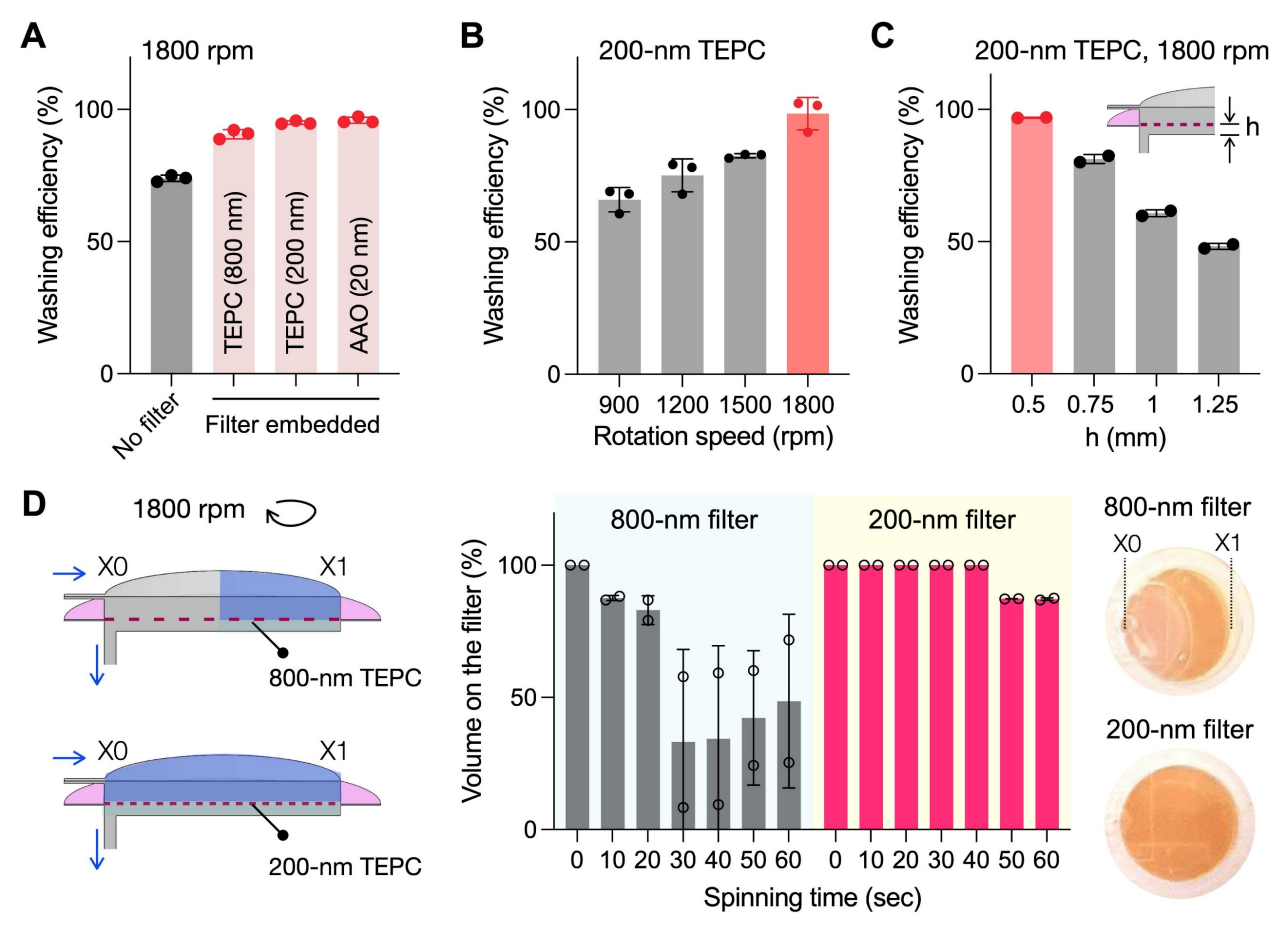
Experimental validation of washing efficiency and volume retention. **(A)** Effect of filters with varying hydraulic resistance. Four different conditions were compared: 800 nm and 200 nm track-etched polycarbonate (TEPC), 20 nm anodic aluminum oxide (AAO), and a control without a filter. Residual dye intensity was measured after washing, where lower intensity indicates improved washing efficiency. Filters with higher hydraulic resistance showed enhanced washing performance. Data are displayed as mean ± s.d. from technical triplicates. **(B)** Effect of rotational speeds. Increasing the rpm improved washing efficiency. Data are displayed as mean ± s.d. from technical triplicates. **(C)** Effect of the chamber height beneath the membrane. Reducing the chamber height (h) below the membrane enhanced washing performance. Data are displayed as mean ± s.d. from technical duplicates. **(D)** Effect of filter hydraulic resistance on fluid retention above the membrane. (Left) Schematic of the filter chamber configurations with different filter pore sizes. (Right) Time-lapse monitoring of the remaining fluid volume above the filter during dye washing. The filter with low resistance failed to retain the fluid. Photographs showed the retained fluid on the membrane after washing. Data are presented as mean ± s.d. from technical duplicates.

At a constant rotation speed of 1800 rpm, washing efficiency improved with membrane hydraulic resistance (**Figure 3A**). Concurrently, under conditions of fixed hydraulic resistance, higher rotational speed resulted in enhanced washing performance (**Figure 3B**). These experimental findings were consistent with simulation predictions, which indicated stronger Coriolis-induced transverse flow patterns with increased rotation speeds (**Figure S6**). We also changed the chamber height beneath the membrane (**Figure 3C**). Reducing this height decreased the residual fluid volume and contaminant accumulation after washing steps, improving the overall washing efficiency.

We further monitored the fluid level of the assay chamber during disc spinning (**Figure 3D**). Maintaining the chamber filled with buffer is critical for minimizing reagent loss and facilitating the transfer of labeled assay products to the collection chamber. The 800-nm TEPC filter demonstrated approximately 50% fluid retention following washing steps, whereas the 200-nm TEPC filter achieved 90% fluidic filling. Overall, the 200-nm TEPC filter was superior in minimizing washing time while maximizing washing efficiency and fluidic retention; we thus selected this filter for the final RapidEx device design.

In the subsequent process optimization, we compared microbeads of three diameters (0.8, 2.1, and 5 µm) to evaluate their impact on detection sensitivity. StAv-coated beads were incubated with biotinylated EVs and fluorescently labeled for CD63, followed by flow cytometric quantification. The detected signal increased with bead size (**Figure S7**), with 5-µm beads generating the strongest fluorescence and consequently achieving the lowest detection limit among the three tested sizes. This improvement is consistent with the larger EV-capture capacity of bigger beads. Based on these results, 5-µm beads were selected for subsequent experiments. These beads were sufficiently small and strongly overdamped (Stokes number ~10^-4^; see **Supplementary Note**), which minimized the likelihood of creating turbulence, local recirculation, or washing dead zones within the chamber.

We also evaluated the impact of surface treatment on bead collection efficiency. Treating the disc interior with an amphiphilic surfactant (1% Pluronic F-127) improved surface hydrophilicity (**Figure S8A**). This treatment, in turn, reduced surface tension and mitigated bead adhesion to the chamber surface. As a result, bead collection efficiency increased dramatically, reaching nearly 100% in the treated chambers (**Figure S8B**).

### Development of a sensitive EV-protein assay

We used the optimized RapidEx system to establish an EV-protein assay. Following EV isolation by size-exclusion chromatography and biotinylation, all subsequent steps were performed entirely within the RapidEx device, including EV capture on microbeads, antibody labeling, and tyramide-assisted signal amplification (see **Table S1** for the details of the disc operation). On-target samples were labeled with marker-specific antibodies, whereas control samples received isotype-matched IgG antibodies (see **Table S2** for antibody information). The total assay time was about 2.1 hours (**Table S4**), substantially shorter than that of a comparable manual TSA-based workflow (12 hours) 10 or a conventional ELISA (6 hours; **Methods**).

We first evaluated whether disc-induced forces affect EV structure. EVs were subjected to the full RapidEx workflow in the presence of surface-blocked microbeads (to prevent EV capture and allow subsequent recovery). Transmission electron microscopy showed that RapidEx-processed EVs retained their characteristic morphology (**Figure S9A**) after the device operation. Immuno-gold staining further confirmed the presence of CD63 on the vesicle membrane (**Figure S9B**), demonstrating that the RapidEx workflow is compatible with downstream protein analysis.

**Figure 4:**
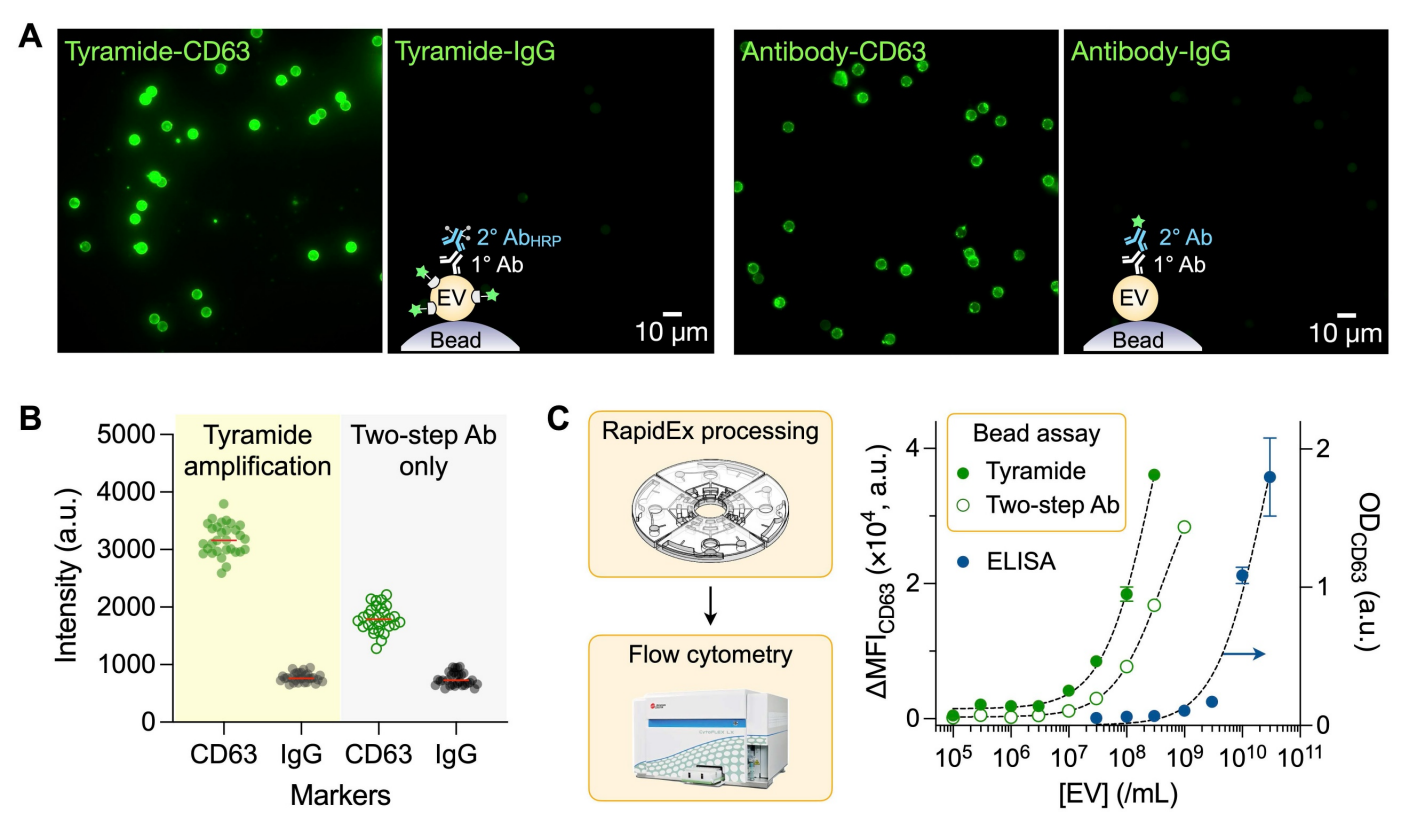
** RapidEx assay development**. **(A)** Fluorescent microscopy confirmed that the RapidEx device assay showed an amplified signal compared to the two-step antibody method, while maintaining minimal background signal in the isotype control. **(B)** Quantification of fluorescent intensity from EV-bead complexes. The mean CD63 signals were 3195 for RapidEx and 1796 with the two-step protocol (1796). The IgG background levels were similar (778 for RapidEx and 749 for the two-step method). Data are from 28 individual beads. **(C)** The titration curve for CD63, measured by flow cytometry, showed that the tyramide-amplified (RapidEx) assay exhibited superior sensitivity compared to the two-step antibody method or traditional ELISA. The estimated detection limits were 4.6 × 10^5^ EVs/mL for the RapidEx device, 1.2 × 10^7^ EVs/mL for the two-step antibody method, and 2.4 × 10^8^ EVs/mL for traditional ELISA. Data are displayed as mean ± s.d. from technical triplicates. The detection limit of the RapidEx device was over 500-fold lower than that of ELISA.

We next compared the fluorescent signals of EV-bead complexes prepared through the tyramide-amplification (RapidEx process) and the conventional two-step antibody labeling method. Fluorescent microscopy confirmed that the RapidEx assay produced strong fluorescence signals for the target protein (CD63) while maintaining minimal background signal from the isotype IgG control (**Figure 4A**). Quantitative intensity analysis further supported this enhancement (**Figure 4B**): the mean CD63 signal (*I*_CD63_) was ~1.8-fold higher with RapidEx (3195) than with the two-step protocol (1796), whereas IgG background (*I*_IgG_) remained similar (778 for RapidEx versus 749 for the two-step method). Consequently, the target-to-background ratio [= (*I*_CD63_ - *I*_IgG_)/*I*_IgG_] improved from 1.4 (two-step) to 3.1 (RapidEx). Stringent washing was critical, particularly for the tyramide amplification, in achieving high signal contrast (**Figure S10**).

We subsequently employed FCM for high-throughput detection of EV-bead complexes. Marker expression was quantified as the difference in median fluorescent intensity (MFI) between targeted and control samples (see **Methods**). EV titration experiments (**Figure 4C**) established a detection limit of 4.6 × 10⁵ EVs/mL for the RapidEx preparation. Assuming that about 12 CD63 molecules are present per EV, this sensitivity corresponds to 10 fM of protein 30, which is consistent with the typical fM-level sensitivity reported for tyramide-amplified ELISA. This detection limit was >25-fold lower than that achieved with two-step antibody labeling of EV-bead complexes (1.2 × 10⁷ EVs/mL) and >500-fold lower than that of conventional ELISA (see **Table S5** for comparison with other systems for EV-protein detection). RapidEx also demonstrated excellent reproducibility, with intra-batch coefficients of variation (CVs) across units ranging from 4% to 9%, and an inter-batch CV of 4% among three different discs (**Figure S11**).

### RapidEx assay of clinical samples

Using the RapidEx device, we evaluated the feasibility of EV-protein profiling for the detection and disease stratification of OvCa. We selected a panel of EV-associated markers commonly reported in human OvCa and supported, when available, by evidence of functional involvement in OvCa pathogenesis: CD24 4, 31, EpCAM 4, 31, FOLR1 32, 33, CLDN3 4, CD44 34, IL6R 35, 36, CLDN4 37, 38, CA125 4, 39, and STn 40, 41. CD63 served as the reference EV marker 42, and expression levels of cancer-associated markers were normalized to CD63 to account for variations in EV input.

A total of 82 plasma samples were analyzed, including healthy female donors (*n* = 28), early-stage high-grade serous OvCa (FIGO I/II; *n* = 16), and late-stage high-grade serous OvCa (FIGO III/IV; *n* = 38). Plasma samples from late-stage patients were collected prior to therapy initiation and retrospectively classified as platinum-sensitive (*n* = 19) or platinum-resistant (*n* = 19) based on treatment response and time to recurrence (see **Table S6** for the patient summary).

**Figure 5:**
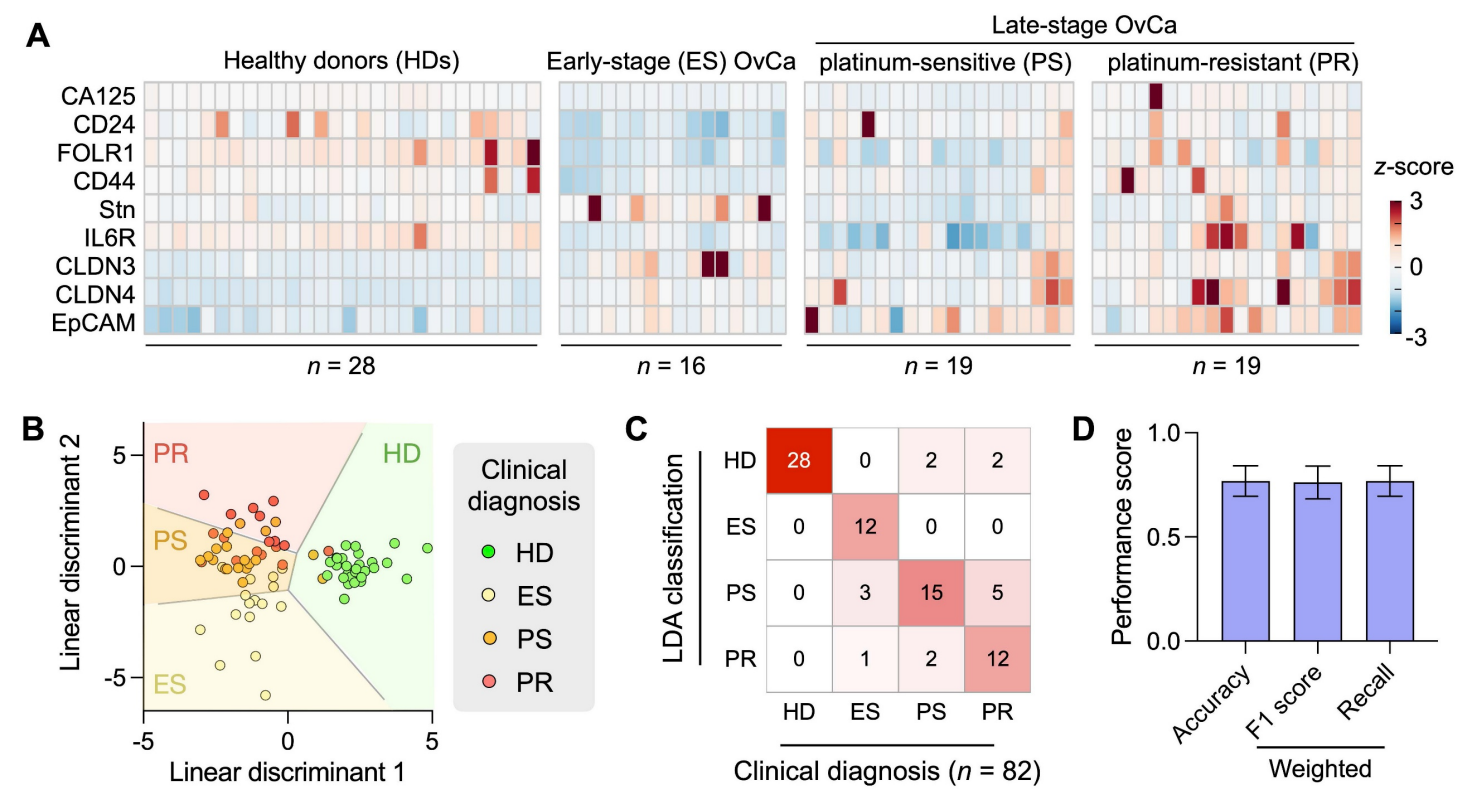
** Clinical application of the RapidEx device for EV-protein profiling in ovarian cancer (OvCa). (A)** EVs were isolated from human plasma samples and immunolabeled within the RapidEx device for OvCa-related protein targets. The heatmap shows EV-protein profiling results for nine markers across healthy donors (HD, *n* = 28), early-stage (ES) OvCa patients (FIGO I/II; *n* = 16), platinum-sensitive (PS) late-stage OvCa patients (FIGO III/IV, *n* = 19), and platinum-resistant (PR) late-stage OvCa patients (FIGO III/IV, *n* = 19). **(B)** Linear discriminant analysis (LDA) was applied to the full marker panel, classifying samples into four groups: HD, ES OvCa, PS, and PR. **(C)** The confusion matrix of the LDA model. The overall classification accuracy was 0.82. **(D)** Leave-one-out cross-validation yielded the accuracy of 0.77, a weighted F1 score of 0.76, and a weighted recall of 0.77. The Bootstrap resampling method (1000 iterations) was used to estimate the standard deviations.

RapidEx-based EV profiling (**Figure 5A**) revealed distinct expression patterns across healthy donors, early-stage OvCa, and late-stage OvCa. Notably, three markers (CLDN3, CLDN4, and EpCAM) demonstrated strong discriminatory power for OvCa diagnosis. This three-marker combination effectively distinguished early-stage OvCa from healthy donors, achieving an AUC of 0.980 in receiver operating characteristic (ROC) analysis (**Figure S12A**). Between all OvCa patients and healthy donors, the three-marker panel maintained similarly high classification performance (AUC = 0.980; **Figure S12B**).

To further assess disease stratification, we applied LDA using the full marker panel. The model successfully classified samples into four clinically relevant groups: healthy donors, early-stage OvCa, platinum-sensitive late-stage OvCa, and platinum-resistant late-stage OvCa (**Figure 5B**). Overall classification accuracy was 0.82, as evaluated by the confusion matrix (**Figure 5C**). Leave-one-out cross-validation (**Figure 5D**) yielded slightly reduced but still strong performance (accuracy = 0.77; weighted F1 = 0.76; weighted recall = 0.77), supporting the robustness of the model.

## Conclusions

We developed the RapidEx platform, which integrates tyramide signal amplification for sensitive protein detection. The device design enables efficient washing through flow redirection driven by centrifugal and Coriolis forces, resulting in substantially reduced background and improved analytical performance. These capacities enabled RapidEx to achieve markedly higher sensitivity than both the conventional bead-based assay and standard ELISA. Such improvement is expected to be important for detecting tumor-derived EVs, which represent a small portion (<5%) of the circulating EV population 18. The increased sensitivity could also provide practical advantages for processing volume-limited samples, including biobanked aliquots and clinical specimens that are often apportioned into small aliquots for multiplexed analysis.

Besides providing enhanced analytical sensitivity, RapidEx addresses several key barriers that have limited the clinical translation of microfluidic-based immunoassays. The workflow is fully automated, self-contained, and programmable, minimizing manual handling during critical steps such as EV capture, washing, and signal amplification; this automation improves assay robustness, reduces operator-to-operator variability, and enhances overall reproducibility. Importantly, RapidEx produces bead-EV complexes that are directly compatible with standard clinical detection instruments (*e.g.*, flow cytometers, fluorescence microscopes, plate readers), enabling seamless integration into existing laboratory workflows. Furthermore, the disc design is compatible with injection molding, allowing for low-cost, mass production of disposable cartridges for routine use.

Our study showed that integrating a low-permeability membrane into a rotating microfluidic platform fundamentally alters the flow behavior by directing fluid laterally rather than vertically through the membrane. In rotating systems, the Coriolis force emerges as an apparent force that deflects moving fluids perpendicular to their direction of motion, in contrast to the centrifugal force. While the role of Coriolis force in enhancing mixing in rotating microfluidic systems has been reported 43, its influence on flow behavior and washing efficiency in membrane-integrated discs has not been systematically characterized. Despite growing interest in such integrated systems 44, detailed mechanistic studies remain limited. In particular, how the membrane modulates the velocity profile under rotation remains largely unexplored, posing challenges in optimizing assay performance in such platforms. Through numerical simulations, we revealed how Coriolis-induced lateral flow, when coupled with membrane resistance, reshaped the velocity field to enhance washing efficiency. We also found that increased disc speed and reduced chamber height beneath the membrane improved the removal of residual substances. Moreover, these optimized conditions helped prevent premature fluid loss during processing by increasing volume retention within the assay chamber. These findings highlighted the importance of a comprehensive design strategy, where rotational speed, hydraulic resistance of the membrane, chamber geometry, spatial positioning, and surface characteristics collectively influence flow behavior.

Given the high mortality and frequent late-stage diagnosis of OvCa, particularly high-grade serous OvCa, a critical need remains for minimally invasive tools for early detection and therapeutic planning 45. Platinum resistance, whether acquired or primary, remains a major clinical challenge, with poor response rates to second-line therapies and limited biomarkers to guide treatment 46. In a proof-of-concept study, we applied RapidEx to process clinical samples and performed EV assays to classify non-cancer subjects, ES OvCa patients, PS late-stage OvCa patients, and PR late-stage OvCa patients. In early-stage OvCa detection, the three-marker panel (EpCAM, CLDN3, and CLDN4) achieved the AUC value of 0.980 in the ROC, which exceeds the AUC value of 0.911 reported for the current clinical standard (longitudinal analysis of serum CA125) 47. More interestingly, probing IL6R expression showed potential to discriminate between PS and PR cases, consistent with previous studies implicating IL-6/IL-6R signaling in treatment resistance and poor prognosis. The ability to detect EVs shed from chemotherapy-resistant subpopulations may offer a means for real-time molecular profiling prior to treatment initiation, addressing a critical unmet need in personalized oncology. However, the relatively small sample size (*n* = 82) represents a key limitation, potentially reducing the discriminative power of the classifier and contributing to modest performance under cross-validation. Further investigations with a larger cohort are necessary to validate the reproducibility of the selected biomarker signals and establish robust statistics.

Looking forward, several key enhancements are envisioned to improve the throughput, sensitivity, and affordability of the RapidEx platform. First, sample throughput can be increased beyond the current four-plex configuration by implementing an on-disc sample-splitting strategy. In this design, a single EV input sample would be partitioned into multiple sub-compartments preloaded with target-specific beads, followed by pan-EV labeling (*e.g.*, CD63) and TSA amplification from shared reagent reservoirs. This parallelized workflow would enable the processing of multiple bead-EV complexes within a single disc operation, substantially increasing throughput without compromising sensitivity. Second, we can incorporate multiplexed signal amplification (see examples in **Figure S13**) and extend detection to intravesicular markers, enabling more comprehensive profiling of EV cargo. Third, we aim to refine Coriolis-enhanced lateral flow by optimizing membrane properties and physical parameters to better match reagent characteristics. For instance, the membrane pore size must be selected to balance hydraulic resistance with reagent permeability, and the membrane thickness must be tuned to ensure a sufficient washing-buffer residence time for robust Coriolis-assisted flow. Systematic optimization of these interdependent factors will help maximize washing efficiency and improve overall assay precision. Collectively, these advances will position RapidEx as a robust and scalable platform for EV-based liquid biopsy and strengthen its utility in precision medicine.

## Supplementary Material

Supplementary figures and tables, movie legends.

Supplementary movie 1.

Supplementary movie 2.

## Data Availability

Source data are provided in this paper. The raw patient datasets generated and analyzed during the study are available from the corresponding authors, subject to approval from the Institutional Review Boards of Dana-Farber / Harvard Cancer.

## Abbreviations

AAO: anodic aluminum oxide
Ab: antibody
AUC: area under the curve
CNC: computer numerical control
CVs: coefficients of variation
ELISA: enzyme-linked immunosorbent assay
Ek: Ekman number
ES: early-stage
EV: extracellular vesicle
FCM: flow cytometry
H_2_O_2_: hydrogen peroxide
HD: healthy donor
IRB: Institutional Review Board
LDA: Linear Discriminant Analysis
LOOCV: leave-one-out cross-validation
M: target marker
MFI: median fluorescent intensity
NTA: Nanoparticle tracking analysis
OvCa: ovarian cancer
PC: polycarbonate
poly-HRP: polymerized horseradish peroxidase
PR: platinum-resistant
PS: polystyrene
PS: platinum-sensitive
ROC: receiver operating characteristic
SEC: Size-exclusion chromatography
StAv: streptavidin
TEM: transmission electron microscopy
TEPC: track-etched polycarbonate
