# Supplementary material for: Engineering inertial flow patterns for signal amplification in disc-based protein assays
Source: Theranostics. 2026 Mar 9;16(10):5226–39. doi: 10.7150/thno.124439 (PMC13080452; doi:10.7150/thno.124439)
Supplement: Supplementary file 1 — Supplementary figures and tables, movie legends. [file thnov16p5226s1.pdf]

SUPPORTING INFORMATION

Engineering inertial flow patterns for signal amplification in disc-based protein assays

Hyun-Kyung Woo[a,b,1], Lauren Philp[c,d,1‡], Dae-Han Jung[a], Dominique Zarrella[c,d], Yein Chung[a,e], Yoonjeong Choi[a,b], Jueun Jeon[a,b], Hyunho Kim[a,b,f], Cesar M. Castro[a,g]*, Bo R. Rueda[c,d]*, Hakho Lee[a,b]*

[a] Center for Systems Biology, Massachusetts General Hospital Research Institute, Boston, MA 02114, USA
[b] Department of Radiology, Massachusetts General Hospital, Harvard Medical School, Boston, MA 02114, USA
[c] Vincent Center for Reproductive Biology, Department of Obstetrics and Gynecology, Massachusetts General Hospital, Boston, MA 02114, USA
[d] Obstetrics, Gynecology and Reproductive Biology, Harvard Medical School, Boston, MA 02114, USA
[e] Department of Immunology, University of Toronto, Toronto, ON M5S 1A8, Canada
[f] School of Mechanical Engineering, Korea University, Seoul, 02841, Republic of Korea
[g] Cancer Center, Massachusetts General Hospital, Harvard Medical School, Boston, MA 02114, USA

‡Present address: Division of Gynecologic Oncology, Department of Obstetrics and Gynecology, University of Toronto, and University Health Network, Toronto, ON M5S 1A8, Canada

[1]These authors contributed equally.

Table of Contents

Supplementary Figures

Figure S1. Characterization of EVs isolated from patient plasma samples.

Figure S2. RapidEx operation steps.

Figure S3. Spinning setup for RapidEx.

Figure S4. Distance-dependent effect.

Figure S5. Effect of viscosity and rotational speed on Coriolis-induced lateral flow.

Figure S6. Correlation between washing efficiency and lateral flow velocity.

Figure S7. Evaluation of bead sizes on the EV assay sensitivity.

Figure S8. Hydrophilic treatment of the disc.

Figure S9. Transmission electron microscopy (TEM) analysis of EV integrity before and after RapidEx processing.

Figure S10. Microscopic images of beads.

Figure S11. Reproducibility assessment.

Figure S12. Diagnostic performance of the three-marker panel (CLDN3, CLDN4, EpCAM) for ovarian cancer (OvCa) detection.

Figure S13. Proof-of-concept multiplexed EV detection.

Supplementary Tables

Table S1. RapidEx operation procedure.

Table S2. List of antibodies used in the current work.

Table S3. Hydraulic resistance based on filter specifications.

Table S4. Overall sample processing in the current report.

Table S5. Comparison of EV detection limits among different sensing systems.

Table S6. Clinical sample information.

Supplementary Movies

Movie S1. Simulation of fluidic flow without a filter.

Movie S2. Simulation of fluidic flow with a filter.

Supplementary Note

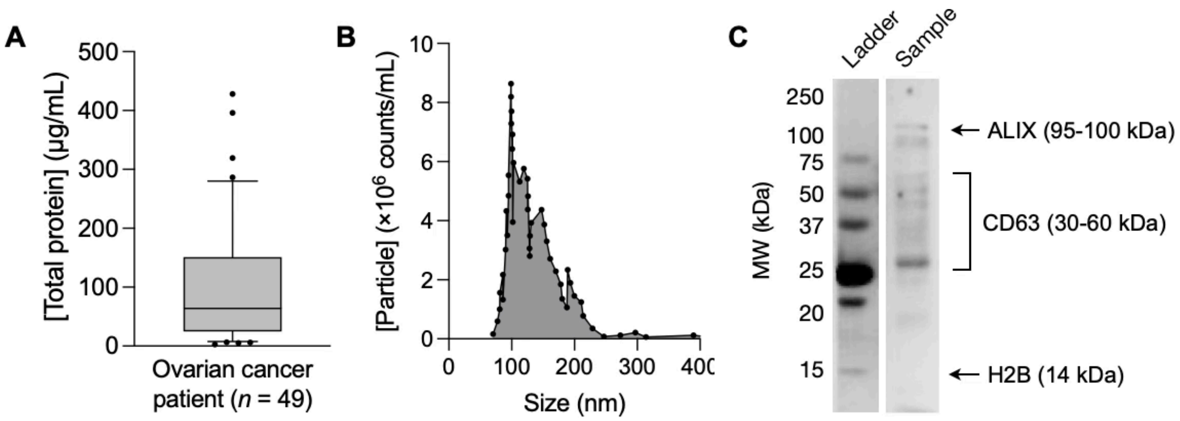

Figure S1. Characterization of EVs isolated from patient plasma samples. (A) Total protein content of isolated EV samples from ovarian cancer patients (*n* = 49). The median value is 63.8 μg/mL. The whiskers indicate the 10th and 90th percentiles. **(B)** Nanoparticle tracking analysis showed the size distribution of isolated particles, with the majority falling within the expected EV size range (50–300 nm). **(C)** Western blot analysis confirmed the presence of canonical EV markers (CD63, a transmembrane protein, and ALIX, an intravesicular protein). The level of the non-EV marker histone H2B (a nuclear protein) was negligible, indicating minimal contamination from cellular or nuclear components.

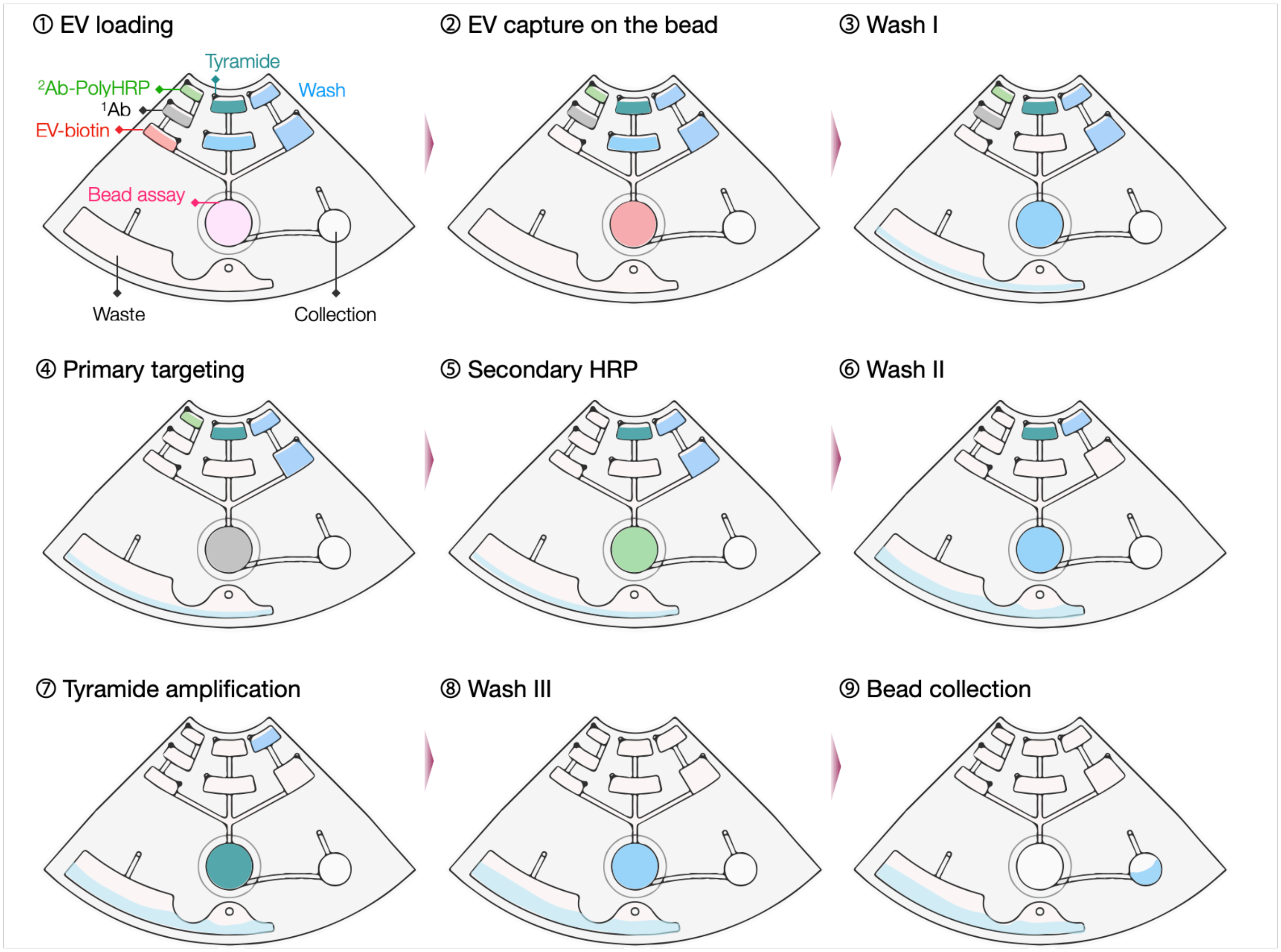

Figure S2. RapidEx operation steps.

① A biotinylated EV (50 μL) sample is loaded.

② EVs are transferred to an assay chamber containing StAv-coated polystyrene beads. EVs bind to the bead surface.

③ EV-bead complexes are washed by introducing a buffer.

④ Primary antibody (1° Ab) against a target protein is introduced to the assay chamber.

⑤ Secondary antibody (2° Ab) conjugated with poly-HRP is introduced to the assay chamber.

⑥ Excess antibody reagent is removed via washing.

⑦ A tyramide working reagent is introduced to the assay chamber.

⑧ Excess tyramide reagent is removed with another washing step.

⑨ The labeled EV-bead complexes are transferred to the collection chamber.

⑩ The collected EV-bead complexes are ready for fluorescent measurements (e.g., microscopy, flow cytometry).

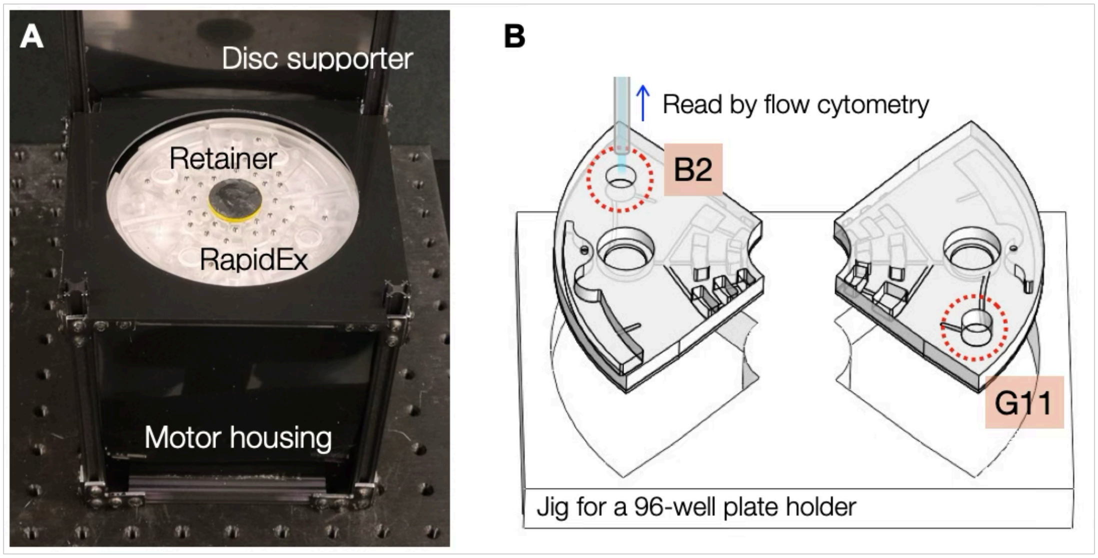

Figure S3. Spinning setup for RapidEx. (A) Photograph of the spinning hardware with the complete disc. The system incorporated a microcontroller that communicated with an external computer for user input. Based on these inputs, the microcontroller regulated the motor speed and spinning time. **(B)** The disc cartridge, which was prepared for the assay, was assembled on a 96-well plate holder jig, and the fluorescence intensity of the beads was measured using flow cytometry.

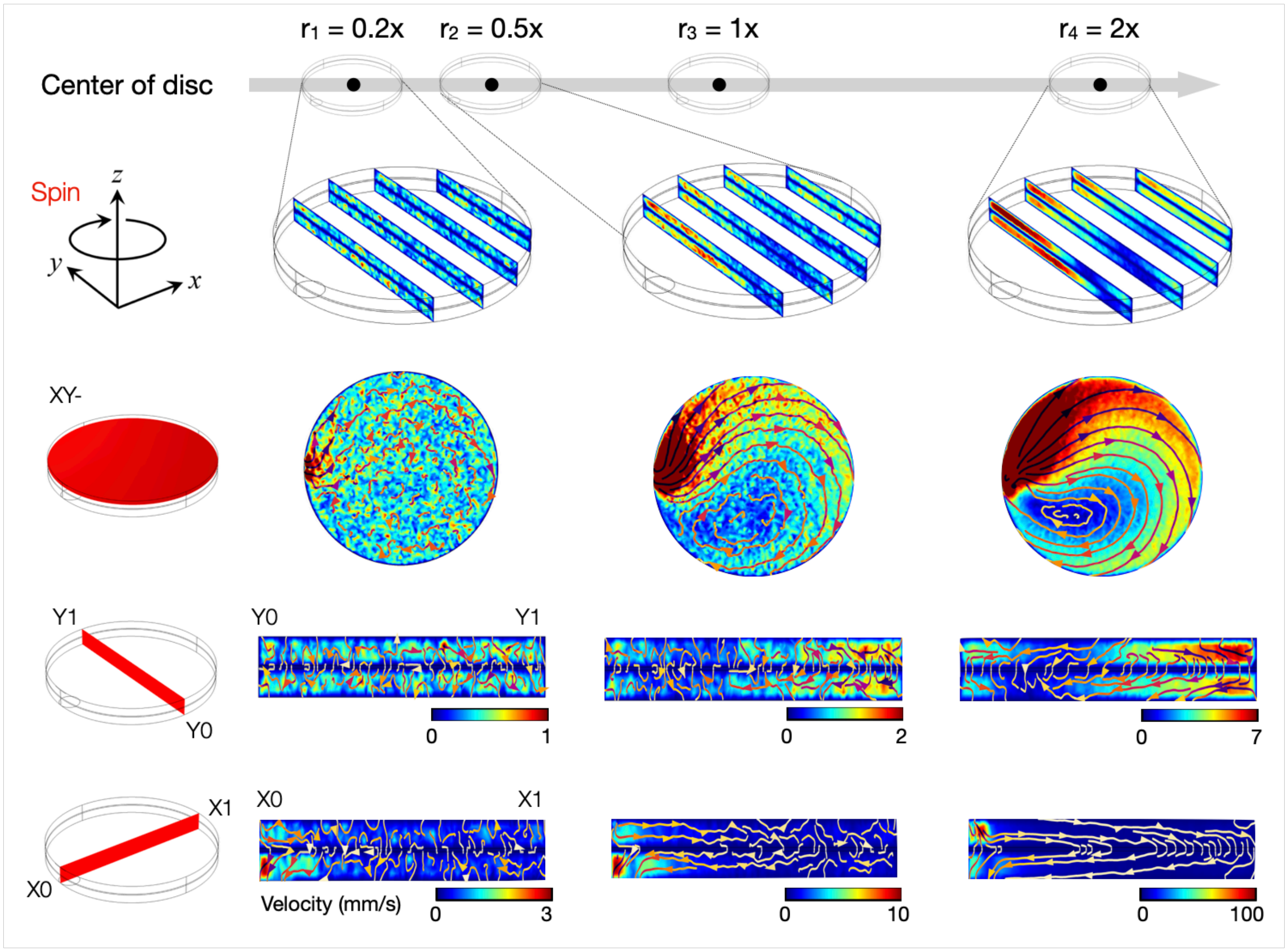

Figure S4. Distance-dependent effect. Numerical simulations showed the flow velocity across different cross-sectional planes (*x*-*y*, *y*-*z*, and *x*-*z* views) for the bead assay chamber equipped with a filter at a rotation speed of 1800 rpm. When the assay chamber was repositioned inward to 20% (r_1, 0.2x) of its original radial distance (r_3, 1x), only limited detouring and mild flow asymmetry were observed. In contrast, placing the chamber at farther radial positions (r_4, 2x) led to stronger detouring flow and the flow deviation.

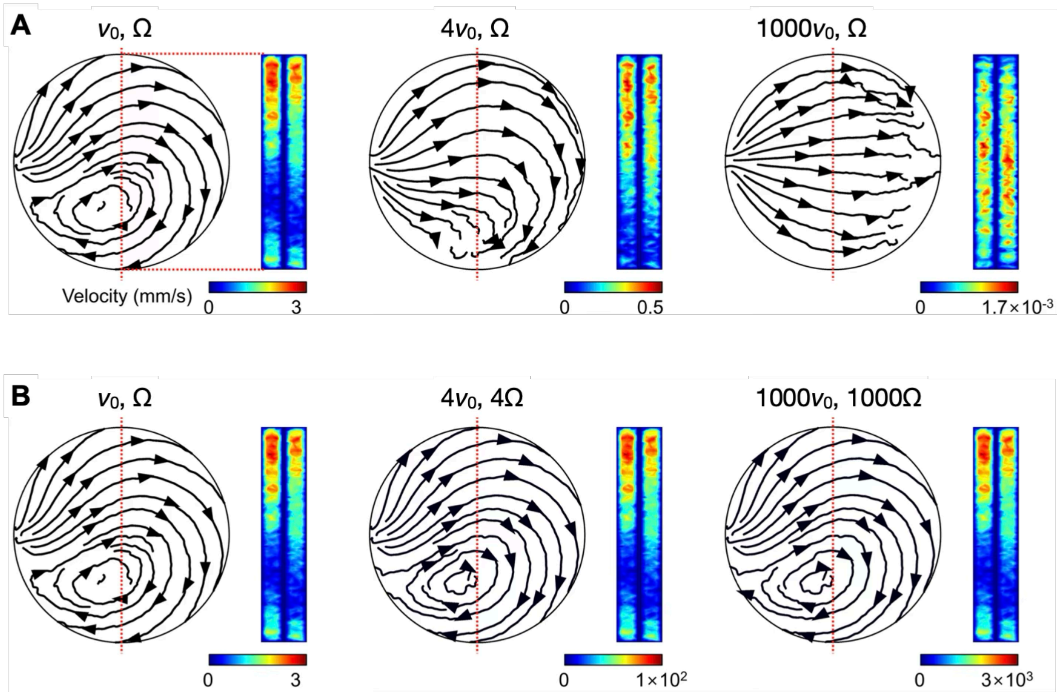

Figure S5. Effect of viscosity and rotational speed on Coriolis-induced lateral flow. (A) Flow fields simulated at the baseline rotational speed (Ω = 1800 rpm) for three kinematic viscosities (v_0, 4v_0, 1000v_0), where v_0 is the kinematic viscosity of water (1.0 mm^2/s). Increasing viscosity (hence, a higher Ekman number, E_k) reduces the lateral deviation of the flow. The chamber height was 0.5 mm. E_k numbers are 2.1×10^{-2}, 8.4×10^{-2}, and 21 (from left to right). **(B)** Proportionally increasing the rotational speed (Ω, 4Ω, 1000Ω) to maintain a constant E_k (2.1×10^{-2}) restores the original flow pattern, demonstrating that Coriolis-driven lateral flow is governed by the inertial–viscous balance captured by E_k.

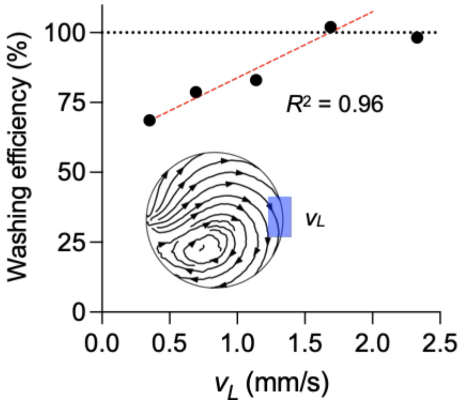

Figure S6. Correlation between washing efficiency and lateral flow velocity. Washing efficiency (η) was experimentally measured under varying rotational speeds, and the corresponding lateral flow velocities (v_L) were obtained from simulated flow fields. A positive linear correlation was identified between these parameters ($\eta = 23.6 \times v_L + 60.2$). The efficiency subsequently reached a plateau when $v_L \geq 1.7$ mm/s. Data points represent mean values obtained from technical duplicates.

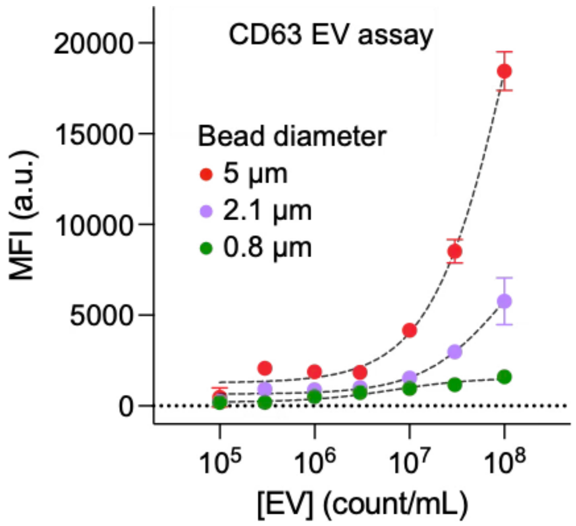

Figure S7. Evaluation of bead sizes on the EV assay sensitivity. Biotinylated EVs were captured on StAv-coated polystyrene beads and fluorescently labeled for CD63 detection. Fluorescence intensity was measured by flow cytometry. Using 5-μm beads yielded the highest sensitivity. The detection limits were 4.6×10^5 EVs/mL (5 μm), 2.6×10^6 EVs/mL (2.1 μm), and 3.3×10^6 EVs/mL (0.8 μm). MFI, median fluorescent intensity. Data are displayed as mean ± s.d. from technical triplicates.

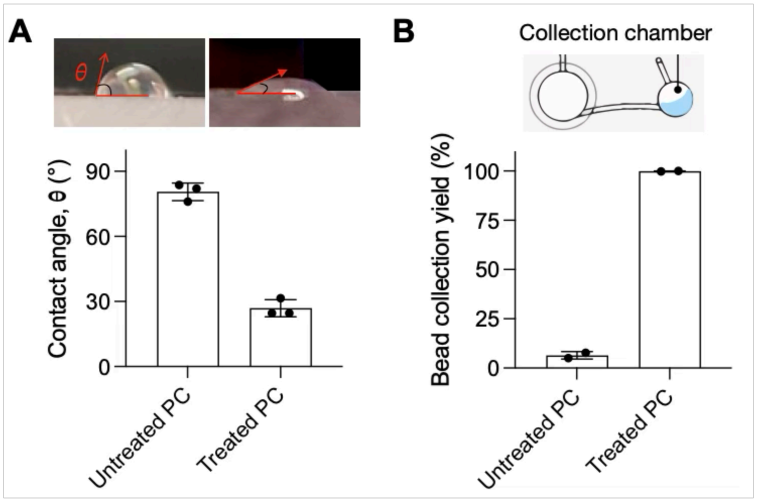

Figure S8. Hydrophilic treatment of the disc. (A) Effect of hydrophilic coating on contact angle. A polycarbonate (PC) was treated with a 1% Pluronic solution (filtered through a 0.22-*μ*m filter). After the treatment, the contact angle of a water drop decreased from 80.6° to 26.9°. Data are presented as mean ± s.d. from technical triplicates. **(B)** Quantitative assessment of bead transfer yield. AF488-conjugated polystyrene beads were used to generate a standard curve. The fluorescence intensities of the input beads and the collected beads in the collection chamber, after transferring the beads from the assay chamber, were measured for both untreated and treated PC devices. Surface treatment markedly improved bead transfer yield, increasing it from <10% to nearly 100%.

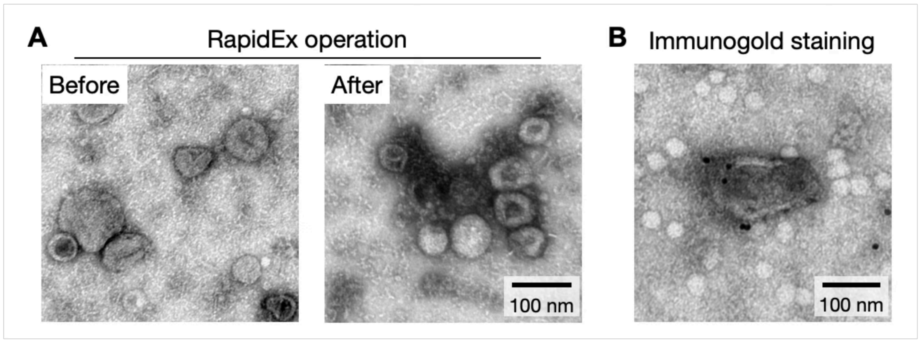

Figure S9. Transmission electron microscopy (TEM) analysis of EV integrity before and after RapidEx processing. (A) Representative TEM images of EVs before (left) and after (right) the RapidEx operation. EVs maintained their characteristic morphology with intact lipid membranes and no signs of structural deformation. **(B)** Immunogold labeling of CD63 on EVs. TEM imaging confirmed the presence of the marker on the vesicle membrane following RapidEx processing. Black dots indicate gold nanoparticles (20 nm in diameter).

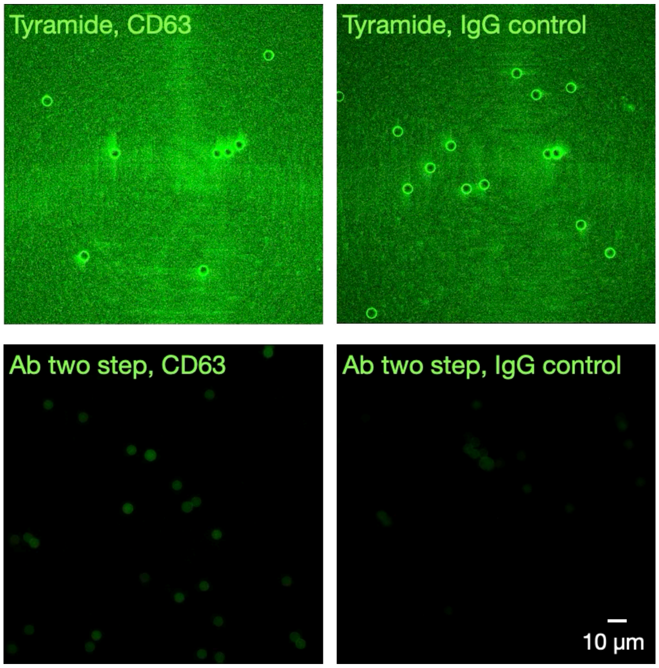

Figure S10. Microscopic images of beads. Under conditions of insufficient washing, the tyramide-amplified assay exhibited increased background signals.

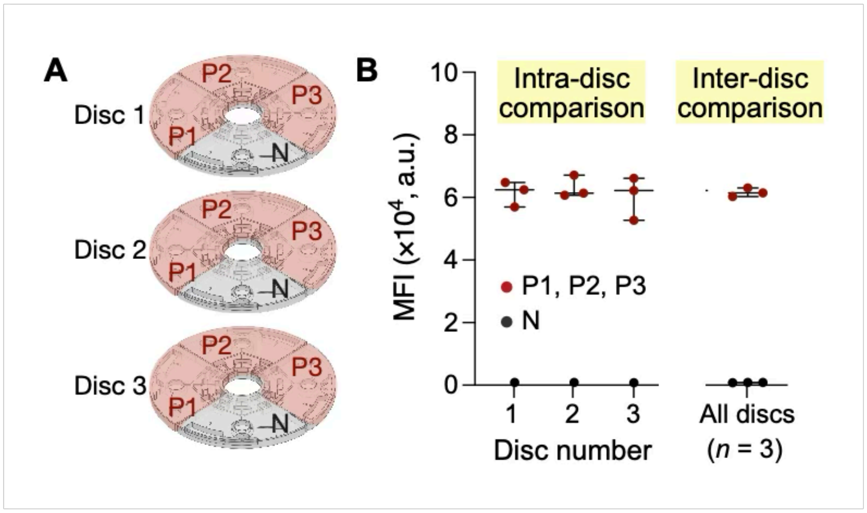

Figure S11. Reproducibility assessment. (A) Schematic representation of three independently fabricated RapidEx discs (Disc 1-3), each containing four assay units. Three units were loaded with a positive sample (P1, P2, P3) containing the target analyte, and one unit served as a negative control (N) without the target. **(B)** The mean fluorescence intensity (MFI) was measured for each unit. For the units P1–P3, the intra-batch coefficients of variation (CVs) were 9.1%, 4.4%, and 8.2% across three discs. The signals from the N units were negligible. The inter-batch CV, assessed by comparing disc-averaged MFIs, was 4%.

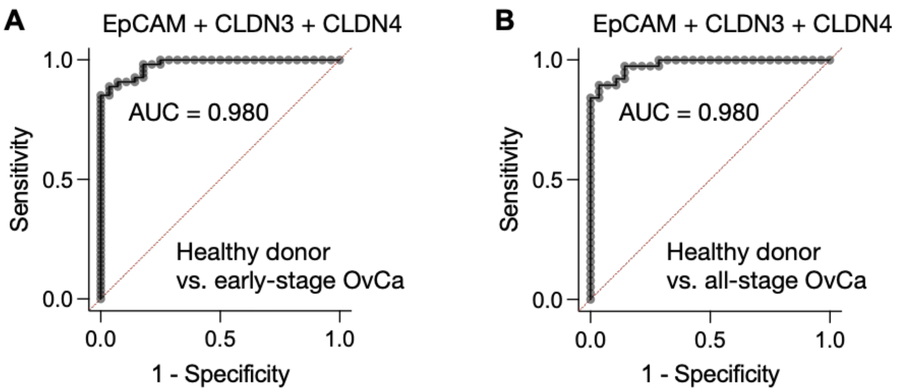

Figure S12. Diagnostic performance of the three-marker panel (CLDN3, CLDN4, EpCAM) for ovarian cancer (OvCa) detection. (A) Receiver operating characteristic (ROC) curve for distinguishing healthy donors from early-stage OvCa patients (FIGO I/II). The three-marker combination achieved an area under the curve (AUC) value of 0.980. **(b)** ROC curve for distinguishing healthy donors from all OvCa patients (combined early- and late-stage). The three-marker combination yielded an AUC of 0.980.

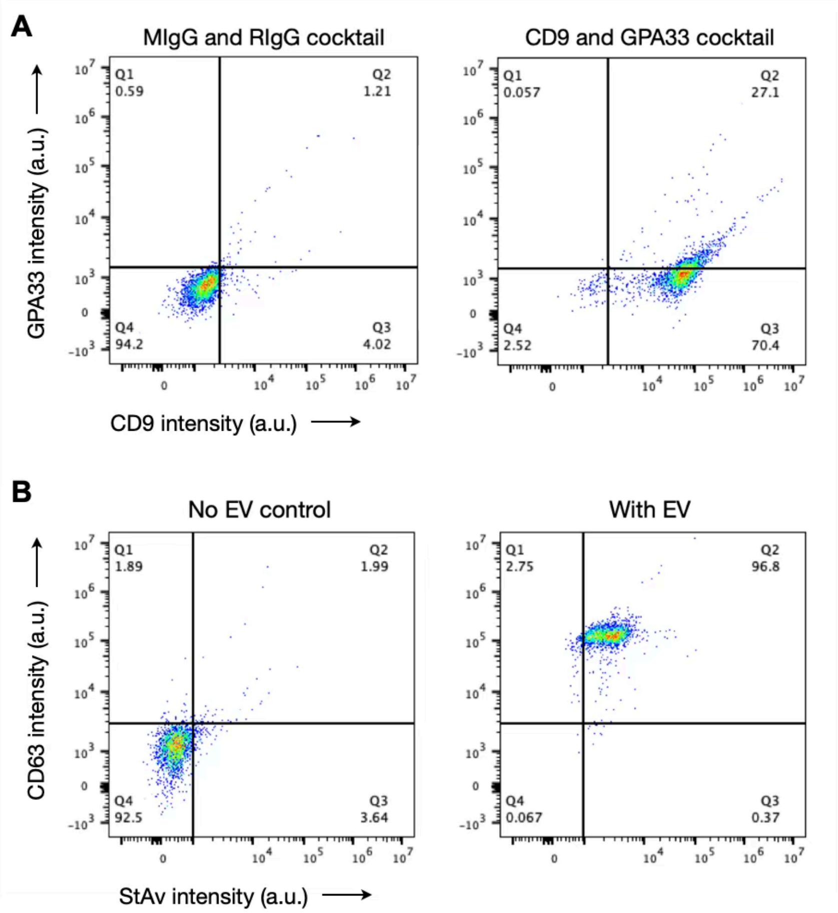

Figure S13. Proof-of-concept multiplexed EV detection. (A) Dual-color marker detection using host-specific antibody labeling. SW620-derived EVs were captured on microbeads and labeled with an antibody cocktail targeting CD9 (mouse IgG) and GPA33 (rabbit IgG), or with a species-matched mouse and rabbit IgG isotype control. Beads were subsequently stained with secondary antibodies (anti-rabbit AF488 and anti-mouse PE). Flow cytometry measurements enabled the simultaneous detection of both markers within the same sample. **(B)** Orthogonal multiplexing using global EV labeling and marker-specific amplification. Biotinylated EVs were captured on microbeads, followed by tyramide signal amplification (TSA) of a target EV protein (CD63) and fluorescent streptavidin labeling (StAv-PE) to report total EV content. Flow cytometry analysis shows distinct fluorescence signals corresponding to global EV abundance and marker-specific amplification.

Table S1. RapidEx operation procedure.

Step	No.	Speed (rpm)	Time (sec)	Operation
EV capture on beads	1	1200	10	EV-biotin transfer
	2	4 rocking modes†	1200	EV capture on StAv-coated beads
	3	1800	60	Wash I
EV protein labeling	4	1200	10	1º Ab transfer
	5	2 rocking modes	600	1º Ab labeling
	6	1200	10	2º Ab-poly HRP transfer
	7	2 rocking modes	600	2º Ab-poly HRP labeling
	8	1800	60	Wash II
Tyramide amplification	9	1200	10	Tyramide working reagents transfer
	10	2 rocking modes	600	Tyramide amplification
	11	1800	60	Wash III
	12	1200	10	Amplified bead transfer
**Total time (min)**			53.8	

†Rocking mode: ±120° oscillation/sec for 30 sec, followed by 50 rpm rotation for 300 sec.

Table S2. List of antibodies used in the current work.

	Marker	Vendor	Host
Primary	CD9	BD bioscience (555370)	Mouse
	CD63	Ancell (215-820)	Mouse
	CD24	eBioscience (14-0247-82)	Mouse
	EpCAM	Abcam (ab187372)	Mouse
	FOLR alpha	RnD systems (MAB5646)	Mouse
	Claudin 3	RnD systems (MAB4620)	Mouse
	CD44	Biolegend (397502)	Mouse
	IL6R	Invitrogen (AHR0061)	Mouse
	Claudin 4	Abcam (ab53156)	Rabbit
	CA125	Abcam (ab693)	Mouse
	STn	SBHsciences (3f1)	Mouse
	GPA33	Sigma aldrich (HPA018858)	Rabbit
Isotype	Mouse IgG1	Bioxcell (BE0083)	Mouse
	Mouse IgG2	Sigma aldrich (M5409)	Mouse
	Rabbit IgG	Abcam (ab172730)	Rabbit
Secondary	Mouse IgG (H+L) poly HRP	Thermofisher (32230)	
	Rabbit IgG (H+L) poly HRP	Thermofisher (B40943)	
Fluorescent label	Tyramide SuperBoost Kit	Thermofisher (B40943)	
	anti-Mouse IgG (H&L) AlexaFluor 488	Thermofisher scientific (A11001)	
	anti-Rabbit IgG (H&L) AlexaFluor 488	Thermofisher scientific (A11008)	
	anti-Mouse IgG (H&L) PE	Thermofisher scientific (P852)	

Table S3. Hydraulic resistance based on filter specifications.

Filter type	Pressure drop (Δp, kPa)	Water flow rate (cm/s)	Membrane thickness (μm)	Permeability (κ, m^2)
800 nm TEPC[1]	69	1.5	9	1.96×10^{-15}
200 nm TEPC[1]	69	0.167	10	2.42×10^{-16}
20 nm AAO[2]	414	0.038	60	5.44×10^{-17}

[1] https://www.sterlitech.com/hydrophilic-polycarbonate-membrane-filter-pct12013100.html

[2] https://www.tischscientific.com/whatman/membranes-inorganic-membranes-anopore-6809-7003

Table S4. Overall sample processing in the current report.

Step	Time (min)	Operation
EV isolation	20	Size exclusion chromatography (SEC) column
	5	Enrichment using a Amicon Ultra-2 centrifugal filter
	40	EV biotinylation
RapidEx process	21	EV capture on beads
	21	EV protein labeling
	11	Tyramide amplification
Flow cytometry	5	Signal measurement
**Total time (hrs)**	2.1	

Table S5. Comparison of EV detection limits among different sensing systems.

Sensing system / platform	Assay time (hr)	Limit of detection (EV/mL)	Reference
Conventional ELISA for EVs	6	~1×10^7	1,2
Electrochemical sensing	2	~ 1×10^4	1,2
Plasmonic sensor	3	3×10^3	3
Microfluidic fluorescence assay	Not available	5.7×10^5	4
Electrochemiluminescence (ECL)	2	1.5×10^4	5
Thermophoretic aptasensor	1	3.8×10^7	6
TSA ELISA for EVs	11	2.4×10^4	7
Electrochemical Impedance	2	5×10^2	8
RapidEx (this work)	2	4.6×10^5	This work

1. Jeong, S. et al. Integrated Magneto-Electrochemical Sensor for Exosome Analysis. *ACS Nano* **10**, 1802-1809 (2016).
2. Park, J. et al. An integrated magneto-electrochemical device for the rapid profiling of tumour extracellular vesicles from blood plasma. *Nat. Biomed. Eng.* **5**, 678-689 (2021).
3. Im, H. et al. Label-free detection and molecular profiling of exosomes with a nano-plasmonic sensor. *Nat. Biotechnol.* **32**, 490-495 (2014).
4. Zhao, T. et al. Fluorescence Characterization of Extracellular Vesicles Using Single-Molecule Confocal Microscopy. *Small Methods* **9**, e00907 (2025).
5. Cho, Y. K. et al. Discovery of a new coreactant for highly efficient and reliable electrochemiluminescence. *Cell Rep. Phys. Sci.* **6**, 102864 (2025).
6. Tian, F. et al. Protein analysis of extracellular vesicles to monitor and predict therapeutic response in metastatic breast cancer. *Nat. Commun.* **12**, 2536 (2021).
7. Jo, A. et al. Inaugurating High-Throughput Profiling of Extracellular Vesicles for Earlier Ovarian Cancer Detection. *Adv. Sci. (Weinh)* **10**, e2301930 (2023).
8. Kilic, T. et al. Multielectrode Spectroscopy Enables Rapid and Sensitive Molecular Profiling of Extracellular Vesicles. *ACS Cent. Sci.* **8**, 110-117 (2022).

Table S6. Clinical sample information.

Basic characterisctics	Early-stage ovarian cancer (n=16)	Platinum-sensitive ovarian cancer (*n* = 19)	Platinum-resistant ovarian cancer (*n* = 19)
Age, median (range)	63 (47-79)	59 (42-76)	64 (44-76)
Grade, n (%)			
I	0 (0)	0 (0)	0 (0)
II	2 (13)	1 (5)	0 (0)
III	14 (87)	18 (95)	19 (100)
Unknown	0 (0)	0 (0)	0 (0)
FIGO stage, n (%)			
T1	4 (25)	0 (0)	0 (0)
T2	12 (75)	0 (0)	0 (0)
T3	0 (0)	17 (89)	13 (68)
T4	0 (0)	2 (11)	6 (32)
Unknown	0 (0)	0 (0)	0 (0)
CA-125 level (units/mL), median (range)			
At diagnosis	86 (9.1-1564)	528 (9-11829)	1457.5 (185.1-3914)
After initial treatment	8.4 (6-28.8)	9 (2-35)	26.7 (5.3-182)
At first recurrence	65.4 (55-75.8)	83 (11-2153)	176.9 (24.5-972.9)

SUPPORTING MOVIE LEGENDS

Movie S1.Simulation of fluidic flow without a filter. Simulation video showing that, without a filter, the fluid follows the shortest path through the low-resistance region, with distinct flow behaviors during acceleration (ramp-up spin), constant rotation (constant spin), and deceleration (ramp-down spin). Flow velocity in other areas of the assay chamber, such as near the sidewalls, remains low, resulting in limited washing efficiency. The Euler force during acceleration and deceleration induces dynamic shifts in the velocity profile, whereas under constant rotation, the flow stabilizes along a steady path.

Movie S2. Simulation of fluidic flow with a filter. Simulation video showing that, with a filter present, the main flow was redirected away from the shortest vertical path to follow the lateral pressure gradient formed by the centrifugal and Coriolis forces, resulting in persistent lateral flow deviation and sustained asymmetry in the velocity distribution. Again, the Euler force contributed to dynamic flow variations during non-steady rotational states (ramp-up spin and ramp-down spin), amplifying the complexity of flow paths near the inlet.

SUPPLEMENTARY NOTE

1. Estimation of Hydraulic Resistance

When flowing through the porous filter, the laminar flow of a Newtonian fluid obeys Darcy's law. Darcy's law describes the superficial fluid velocity (U_s) through the porous medium as

(1) $$U_s = \epsilon \langle u_{\parallel} \rangle = -\frac{\kappa}{\mu}\frac{\Delta p}{L_{\parallel}},$$

where Δp denotes the pressure drop over the length $L_{\parallel}$ of the medium in the flow direction, ε is the medium's porosity, $\langle u_{\parallel} \rangle$ is the mean velocity of the fluid in the flow direction, κ is the permeability of the porous medium, and μ is the dynamic viscosity of the fluid. The permeability of a filter is calculated using Eq. (1) based on the filter specifications.

Hydraulic resistance (R_h) is then given as:

(2) $$R_h = \frac{\mu \cdot L_{\parallel}}{\kappa \cdot A},$$

where A is the area of the membrane.

2. Estimation of the Stokes Number in RapidEx

To assess whether suspended microbeads perturb the chamber flow, we estimated the **Stokes number** (S_t), a dimensionless quantity defined as the ratio of the particle response time to viscous drag (t_p) and the characteristic fluid transit time (t_f)

(3) $$S_t = \frac{t_p}{t_f}.$$

When $S_t \ll 1$, particles closely follow fluid streamlines with negligible flow disturbance. In contrast, $S_t \gg 1$ indicates that particles can significantly perturb the local flow field.

For a spherical particle of diameter d_p, the Stokes drag force is

(4) $$F_D = 3\pi\mu d_p \Delta v,$$

where $\Delta v = (v_f - v_p)$ is the relative velocity between the fluid (v_f) and the particle (v_p). The particle's equation of motion is given by

(5) $$m_p \frac{dv_p}{dt} = F_D,$$

where m_p is the particle mass. For a spherical particle with a density of ρ_p,

(6) $$m_p = \frac{\pi \rho_p d_p}{6}.$$

Substituting and simplifying yields

(7) $$\frac{dv_p}{dt} = \frac{18\mu}{\rho_p d_p}\Delta v.$$

Thus, the particle response time is

(8) $$t_p = \frac{\rho_p d_p}{18\mu}.$$

The characteristic fluid transit time across the particle is

(9) $$t_f = \frac{d_p}{v_f}.$$

Substituting into the definition of S_t leads to

(10) $$S_t = \frac{\rho_p d_p}{18\mu} \cdot \frac{v_f}{d_p} = \frac{d_p v_f}{18\mu / \rho_p} = \frac{\mathrm{Re}_p}{18},$$

where Re_p is the particle Reynolds number.

Using the experimental conditions for RapidEx, $d_p = 5\ \mu\mathrm{m}$, $\rho_p = 1.05\ \mathrm{g/cm^3}$ (polystyrene beads), $\mu = 1.0 \times 10^{-3}$ Pa·s, and $v_f = 2$ mm/s, we obtain $\mathrm{Re}_p = 1 \times 10^{-2}$ and consequently $S_t = 6 \times 10^{-4}$. This small S_t value indicates that the microbeads are strongly overdamped and follow the fluid streamlines. Under these conditions, the beads do not induce turbulence, local recirculation, or washing dead zones within the chamber.

3. Interplay between the Coriolis force and viscosity

To evaluate when Coriolis-driven lateral flow becomes dominant in the RapidEx chamber, we analyzed the **Ekman number** (E_k), a dimensionless ratio comparing viscous to Coriolis forces:

(11) $$E_k = \frac{\nu}{\Omega h^2},$$

where ν is the kinematic viscosity, Ω the angular velocity, and h the chamber height. When $E_k \ll 1$, Coriolis forces dominate and strong lateral sweeping flows emerge; when $E_k \gg 1$, viscous effects suppress these flows.

Fluidic simulations showed that increasing viscosity at a fixed rotation speed raised E_k and reduced lateral deviation (**Fig. S5A**), indicating stronger viscous damping. When rotation speed was increased proportionally to viscosity so that E_k remained constant, the original lateral-flow pattern reappeared (**Fig. S5B**). These results confirm that Coriolis-induced flow is governed primarily by the inertial-viscous balance (captured by E_k), rather than by viscosity or rotation alone.

In the current RapidEx, $h = 0.5$ mm and $\Omega = 1800$ rpm (188.5 rad/s). Under these conditions, Coriolis-driven lateral flow is maintained for fluids with kinematic viscosities up to ~5× that of water ($\nu_0 = 1.0\ \mathrm{mm^2/s}$ at 20 °C). This range encompasses most biological fluids, including PBS ($\nu \approx \nu_0$), urine ($\nu \approx 1.07\nu_0$), plasma ($\nu \approx 1.3\nu_0$), and saliva ($\nu \approx 1.5\nu_0$). For PBS, the primary fluid used in this work, $E_k = 2.1 \times 10^{-2}$, which confirms that the operation is in the Coriolis-dominated regime. Overall, this analysis provides a general design principle for disc-based fluidics: **maintaining $E_k < 1$** to ensure robust lateral flow across the device.
